# Kinesin Family Member 26A Disrupts DNA-Dependent Protein Kinase Complex Formation to Enhance Chemoradiotherapy Sensitivity in Colorectal Cancer

**DOI:** 10.7150/ijbs.127218

**Published:** 2026-03-17

**Authors:** Mengjie Li, Ningxin Ren, Shaosen Zhang, Hongxia Chen, Ruoqing Yan, Ying Huang, Jing Jin, Linlin Zheng, Shuangmei Zou, Yexiong Li, Wen Tan, Dongxin Lin

**Affiliations:** 1 State Key Laboratory of Molecular Oncology, Department of Etiology and Carcinogenesis, National Cancer Center/National Clinical Research Center for Cancer/Cancer Hospital, Chinese Academy of Medical Sciences and Peking Union Medical College, Beijing 100021, China.; 2 Department of Thoracic Surgery and Oncology, the First Affiliated Hospital of Guangzhou Medical University, State Key Laboratory of Respiratory Disease & National Clinical Research Center for Respiratory Disease, Guangzhou 510120, China.; 3 Department of Radiation Oncology, National Cancer Center/National Clinical Research Center for Cancer/Cancer Hospital, Chinese Academy of Medical Sciences and Peking Union Medical College, Beijing 100021, China.; 4 Department of Radiation Oncology, National Cancer Center/National Clinical Research Center for Cancer/Cancer Hospital & Shenzhen Hospital, Chinese Academy of Medical Sciences and Peking Union Medical College, Shenzhen 518100, China.; 5 Department of Pathology, National Cancer Center/National Clinical Research Center for Cancer/Cancer Hospital, Chinese Academy of Medical Sciences and Peking Union Medical College, Beijing 100021, China.; 6 Sun Yat-sen University Cancer Center, State Key Laboratory of Oncology in South China, Guangzhou 510060, China.

**Keywords:** KIF26A, colorectal cancer, chemoradiotherapy, NHEJ, HDAC

## Abstract

Chemoradiotherapy is the principal approach for treating a wide range of human cancers. However, its therapeutic outcomes in clinical settings are frequently impaired by resistance to tumor chemoradiotherapy. In this study, we demonstrated that kinesin family member 26A (KIF26A) is downregulated in chemoradioresistant colorectal cancers, as revealed by transcriptomic analyses of colorectal cancer tissues and cell lines. Reduced KIF26A levels predicted diminished responsiveness to chemoradiotherapy and unfavorable outcomes in patients with colorectal cancer. Furthermore, lower KIF26A expression was associated with colorectal cancer (CRC) progression, migration, and invasion. This is the first demonstration that KIF26A interacts with Ku70 to suppress the formation of the DNA-dependent protein kinase (DNA-PK) complex, thereby preventing activation of non-homologous end joining (NHEJ) for repairing DNA damage. This makes cancer cells more vulnerable to DNA damage from chemoradiotherapy, thereby enhancing their sensitivity. To address chemoradio-resistance in KIF26A-low-expressing cells, we ascertained that histone deacetylase inhibitors (HDACi) could enhance acetylation of the KIF26A promoter, upregulate KIF26A, and boost the sensitivity of chemoradiotherapy-resistant cells. Thus, our research elucidates the function of KIF26A in the NHEJ repair process and indicates that combining HDACi with chemoradiotherapy may serve as a promising therapeutic modality for colorectal cancer.

## Introduction

Colorectal cancer (CRC) is a disease of significant global importance. It holds the position as the world's third most frequent cancer diagnosis and the third leading contributor to cancer fatalities 1. Chemotherapy and radiotherapy are integral components of CRC treatment, especially for those with advanced or metastatic disease. However, resistance to chemotherapy and radiotherapy remains a major challenge, leading to treatment failure in many cases 2. This suggests that many patients may not benefit significantly from chemoradiotherapy treatment (CRT), highlighting the need for accurate predictive biomarkers. Recently, studies have focused on identifying key factors involved in chemoradio-resistance and enhancing the chemosensitivity of cancer cells through targeted interventions.

One of the central mechanisms underlying this therapeutic resistance lies in the activation of efficient DNA damage repair pathways within cancer cells 3. These pathways enable rapid repair of DNA lesions induced by radiotherapy and chemotherapeutic agents, allowing cells to evade apoptosis and ultimately leading to treatment failure. Among the various DNA damage repair pathways, non- homologous end joining (NHEJ) serves as a major mechanism for repairing DNA double-strand breaks (DSBs) 4.

NHEJ serves as a linchpin in the DNA repair arsenal, indispensable for maintaining genomic stability through effective repair of DSBs. NHEJ takes on special significance during the response to radiotherapy and chemotherapy, because these treatments often induce DSBs as part of their cytotoxic mechanisms 5. The key components of the NHEJ repair process include the DNA-PK complex, comprising the DNA-PK catalytic subunit (DNA-PKcs) and Ku heterodimer (Ku70/Ku80). Dysfunction or dysregulation of these components can lead to impaired NHEJ, resulting in increased sensitivity to DNA-damaging agents and heightened potential for genomic instability 6.

Recent research has underscored the involvement of kinesin family (KIF) members in regulating cancer cells' sensitivity to chemotherapy and radiotherapy. For example, KIF5B, KIF13B, and KIFC3 are involved in the formation and regulation of DSB-capturing nuclear envelope tubules (dsbNETs), which play vital roles in repairing DSBs 7. KIF14 promotes chemoresistance in triple-negative breast cancer via AKT phosphorylation 8, and silencing KIF18B enhances radiosensitivity in sarcoma models 9. These findings underscore the potential of KIF family members as targets for overcoming chemoresistance and improving radiotherapy outcomes.

In our previous genome-wide expression study on 81 rectal cancer patients, we identified 132 differentially expressed genes distinguishing the chemoradiotherapy-sensitive from -resistant groups, with kinesin family member 26A (KIF26A) being highly expressed in the sensitive group 10. Recent studies have linked KIF26A to processes involving cancer progression such as cell division, transport, and microtubule stabilization. Its aberrant expression is observed in various cancers, suggesting its potential to serve as a biomarker and a target for therapeutic interventions 11, 12. In gastric cancer, low KIF26A expression, particularly in cases with lymph node metastasis, indicates a possible tumor suppressor role 11. Similarly, in pancreatic ductal adenocarcinoma (PDAC), KIF26A is involved in microtubule stabilization, which is crucial for cancer cell survival and proliferation 12. However, the clinical potential of KIF26A for the treatment of colorectal cancer and other malignancies remains unknown.

## Materials and Methods

### Study participants and biospecimens

From January 2006 to June 2013, 81 individuals with locally advanced rectal cancer were consecutively enrolled in the Cancer Hospital, Chinese Academy of Medical Sciences, Beijing, as previously described 10, 13. The eligibility criteria were as follows: (1) age ≥ 18 years; (2) newly diagnosed, locally advanced disease (stage T3-4 and/or node-positive [N+]) as assessed by magnetic resonance imaging (MRI) and/or endorectal ultrasound; (3) no evidence of distant metastasis (M0) at initial diagnosis; (4) baseline blood biochemical parameters within normal limits; (5) a Karnofsky Performance Status (KPS) score of ≥ 70; and (6) a life expectancy of more than 6 months. The median age of the cohort was 54 years, with a male-to-female ratio of 2:1 (67% male to 33% female). All patients underwent concurrent preoperative chemoradiotherapy. The radiotherapy protocol consisted of a total dose of 50 Gy delivered in 25 fractions. Concurrent chemotherapy comprised intravenous oxaliplatin administered weekly at a dose of 80 mg/m²and oral capecitabine at a dose of 1600 mg/m²/day. Surgical resection was performed 4-6 weeks after the completion of neoadjuvant therapy. Pathological assessment of the surgically resected specimen was conducted to evaluate the tumor response. Tumor regression was assessed pathologically on the surgical specimen according to the Mandard Tumor Regression Grade (TRG) criteria. Specifically, 30 specimens, including 7 TRG1 and 23 TRG2 cases, were classified as good responders. Thirty-seven TRG3 specimens were considered intermediate responders. Fourteen specimens, comprising 12 TRG4 and 2 TRG5 cases, were designated as poor responders. Disease-free survival (DFS) was measured from the date of surgery to the time of tumor recurrence, death from any cause, or last follow-up. Follow-up was completed for all 81 patients by October 31, 2021, with a median follow-up duration of 125 months. The research protocol received approval from the Institutional Review Board of the Cancer Institute, Chinese Academy of Medical Sciences (IRB No. 23/088-3827), and conformed to the principles of the Declaration of Helsinki (2024). Genome-wide expression profiles generated from these 81 neoadjuvant chemoradiotherapy-treated rectal cancers were archived in the National Genomic Data Center (https://ngdc.cncb.ac.cn/gsub/) under the project ID PRJCA027384. The baseline characteristics of patients stratified by KIF26A expression level were summarized in Supplementary Table S1.

### Antibodies

The anti-DNA-PKcs (phospho S2056) (cat. #ab124918), anti-γH2AX (cat. #ab81299), anti-GAPDH (cat. #ab8245), anti-Ku80 (cat. #ab80592), anti-Ku70 (cat. #ab92450), and anti-Histone H3 (acetyl K9) (cat. #ab32129) antibodies were purchased from Abcam. The anti-DNA-PKcs (cat. #38168) was obtained from Cell Signaling Technology. The anti-mouse IgG antibody (cat. #B900620) was purchased from Proteintech. The anti-KIF26A (cat. #sc-100949) antibody was obtained from Santa Cruz Biotechnology.

### Cell lines and cell culture

Human colorectal cancer cell lines HCT116, RKO, SW620, HT29, SW480, LoVo and the human lung squamous carcinoma cell line H520 were obtained from the Cell Bank of the Basic Medical College of Peking Union Medical College. Chemotherapy-resistant HCT116 cells were purchased from IMMOCELL (Xiamen, China). HCT116 chemoradiotherapy-resistant cells, referred to as HCT116-R, were established in this study. The methodology for their construction is described in the next section. HCT116, RKO, SW620 and HT29 cells were maintained in high-glucose DMEM (Corning; cat. #10-013-CV) containing 10% or 15% fetal bovine serum (FBS). SW480, LoVo and H520 cells were maintained using RPMI-1640 (Corning; cat. #10-040-CV) supplemented with 10% FBS. All cells were incubated at 37 °C in a 5% CO_2_ atmosphere and regularly treated with mycoplasma-removal reagents. To ensure experimental integrity, we routinely screened all cell lines for mycoplasma contamination with the Universal MycoBlue Mycoplasma Detector (Vazyme; cat. #D101-01). Only mycoplasma-free cell cultures were included in experiments.

### Establishment of chemoradiotherapy-resistant cells

To establish a combined chemoradioresistance model, Oxaliplatin-resistant HCT116 cells (purchased from IMMOCELL, Xiamen, China) were used. These cells were maintained in culture medium containing 2 μM Oxaliplatin to sustain chemoresistance. To confer additional resistance to ionizing radiation, these Oxaliplatin-resistant cells were subjected to a fractionated irradiation protocol. Cell expansion was performed using 10 cm dishes and subjected to 2 Gy of irradiation at 60-70% confluence using the MultiRad225 device. On day 2, a single 2 Gy dose was administered at a rate of 3.5 Gy per minute. After irradiation (IR), cells were returned to the incubator. On day 4, cells were trypsinized, replated in 10 cm dishes, and irradiated again in the same manner. The total accumulated dose reached 60 Gy. Cell viability was confirmed microscopically before proceeding with further experiments.

To validate the successfully established chemoradioresistant phenotype, the generated cell line and the parental, non-resistant control cell line were subjected to a series of sensitivity assays. This included extreme limiting dilution assays, IC_50_ determination, and colony formation assays under combined chemoradiotherapy treatment. For long-term maintenance of resistance, cells were kept in 2 μM Oxaliplatin and irradiated weekly with 2 Gy.

### Establishment of cell lines overexpressing and knocking down KIF26A

The lentivirus for stable overexpression of KIF26A was purchased as viral particles from GeneChem (Shanghai, China). The lentivirus for stable knockdown of KIF26A was purchased from GenePharma (Suzhou, China). Lentivirus transfection was performed using the specified reagent. After incubation in complete medium for ~24 hours, cells were subjected to selection in complete growth medium supplemented with 2-4 μg/mL puromycin for at least one week. Stable overexpression (OE) and knockdown (sh) of KIF26A were assessed by reverse transcription quantitative PCR (RT-qPCR) and Western blotting (WB). The shRNA sequences are available in Supplementary Table S2.

### siRNA transfection of cells

Cells in viable condition were seeded into 6-well plates with thoroughly mixed suspensions and cultured overnight at 37 °C. We replaced the medium with 1 mL of Opti-MEM (Gibco; cat. #31985070) once the cells reached ~30% confluence. Transfection complexes were formulated by separately incubating Lipofectamine 2000 (5 µL; Invitrogen; cat. #11668500) and siRNA (5 µL of 20 µM stock), each with 250 µL Opti-MEM, for 5 minutes at room temperature, respectively. The two solutions were then combined and incubated for 20 minutes at room temperature. The complexes were added dropwise to the wells. After 6 hours, the Opti-MEM with siRNA was replaced with 2 mL complete medium for continued culture. Knockdown (KD) efficiency was verified by RT-qPCR. The siRNAs were ordered from GenePharma (Shanghai, China). See Supplementary Table S3 for sequence details.

### Cell viability assays

Cells were seeded in 96-well plates at appropriate densities. On day 2, plates were randomly grouped and subjected to different treatments. Cell viability was assessed every 24 hours. CCK-8 reagent (Dojindo Lab; cat. #CK04) was diluted 1:10 in serum-free medium, incubated with the cells at 37 °C for 1.5 hours, measuring the absorbance at 450 nm. To determine half-maximal inhibitory concentration (IC_50_), cells were exposed to different concentrations of Oxaliplatin. After 48 hours, CCK-8 absorbance was measured, and IC_50_ values were determined by nonlinear regression analysis. Each experiment included six replicate wells.

### Colony formation assays

Low-density cultures were established in six-well plates. On day 2, plates were randomly grouped and subjected to different treatments. After 10-14 days, colonies were fixed using 4% paraformaldehyde (Aoqing Biotech; cat. #AQ-201-500 mL) and stained with crystal violet solution (Beyotime; cat. #C0121-100 mL). Each experiment had three biological replicates. Colonies were quantified using ImageJ software.

### Extreme limiting dilution assays

Cells were plated in 96-well plates under viable growth conditions. A suspension of 2 × 10^4^cells in 10 mL medium was prepared. Five milliliters of this suspension was added to 5 mL of medium and serially diluted 2-fold to generate six tubes. Each tube was used to plate 20 replicate wells in two separate plates with 200, 100, 50, 25, 12, and 6 cells per well. Plates were incubated at 37 °C overnight. One plate was subjected to 2 Gy IR. After 14 days, cells were fixed with methanol, stained with crystal violet solution (Beyotime; cat. #C0121-100 mL), and wells with colonies were counted. The ELDA website (https://bioinf.wehi.edu.au/software/elda) 14 was used to analyze group differences and generate statistical results.

### Transwell assays

For the migration assay, serum-starved cells were plated in the upper chamber (Corning; cat. #3422) and incubated for 18 hours, with the lower chamber containing medium with 10% serum. For the invasion assay, Matrigel (Corning; cat. #354234) was first applied to the upper chamber, and cells were maintained in serum-free medium and incubated for 26 hours with the lower chamber containing medium with 15% serum. Following incubation, cells were fixed and stained with crystal violet solution (Beyotime; cat. #C0121-100 mL) for 1 hour. Each experiment had three biological replicates, and migrated cells were counted with ImageJ.

### Quantitative real-time PCR

We isolated RNA using the RNA Rapid Purification Kit (ES Science; cat. #EZB-RN001-plus) and then performed reverse transcription with PrimeScript RT Master Mix (Takara; cat. #RR0036A). Quantitative real-time PCR analysis was conducted using the SYBR Premix Ex Taq II kit (Takara; cat. #CN830S) on a Q225 Quantitative PCR instrument with a 96-well block (KUBO; cat. #q225-PLATE-10). We employed the ΔΔCt method to normalize gene expression data to GAPDH and assess relative changes across groups. See Supplementary Table S4 for primer sequences.

### Western blot analysis

Cells were washed twice with ice-cold PBS and subsequently lysed in RIPA buffer (Beyotime; cat. #P0013B) supplemented with 1× phosphatase inhibitor cocktail (NCM Biotech; #P002). After 25-minute incubation in a 4 °C metal bath and centrifugation at 14,000 × g for 15 min at 4 °C, supernatants were collected from lysates and protein concentrations were measured with a BCA kit (Thermo Fisher Scientific; cat. #1863381). Samples were mixed with SDS-PAGE lading buffer (NCM Biotech; cat. #WB2001) and heated to 95 °C for 15 minutes. Equal volumes were loaded on SDS-PAGE gels along with molecular weight markers (Thermo Scientific; cat. #26617). Proteins were electrotransferred to PVDF membranes (Millipore, Burlington, MA, USA) via wet transfer. After blocking with 5% skimmed milk in 1× TBST (TBS + 1% Tween-20) for 2 hours at room temperature, membranes were incubated with primary antibodies in universal diluent (NCM Biotech; cat. #WB500D) overnight at 4 °C. Following three TBST washes (10 minutes each), HRP-conjugated secondary antibodies were applied for 2 hours at room temperature. After three additional washes, membranes were developed using ECL reagent (MeilunBio; cat. #MA0186), and signals were captured using the Amersham Imager 600. Each Western blot analysis was performed with three biological replicates.

### RNA-seq analysis

Transcriptome profiling was conducted using the Illumina NovaSeq 6000 platform (Tsingke Biotechnology). Differential expression was analyzed using DESeq2, with significance defined as adjusted *P* < 0.05 and fold-change thresholds of ≤ 0.5 or ≥ 2.

### Multiple immunofluorescence assays

HCT116 and RKO cells were grown to 40-50% confluence on coverslips (CITOTEST; cat. #80346-0910). Following PBS washes, cells were fixed in ice-cold 4% paraformaldehyde for 10 minutes, permeabilized with 0.1% Triton X-100 (Sigma-Aldrich; cat. #T9284) for 6 minutes, and blocked with 3% bovine serum albumin (Beyotime; cat. #ST023). Diluted primary antibodies were applied overnight at 4 °C. After PBS washes, Alexa Fluor secondary antibodies were added for 30 minutes at room temperature, protected from light.

When both primary antibodies were from the same species, one antibody and its secondary were incubated first, followed by PBS washes and then the other primary antibody and its secondary. Coverslips were washed again, mounted with DAPI-containing mounting medium (ZSGB-BIO; cat. #ZLI-9557), and imaged using a confocal microscope (AKOYA). Immunofluorescence intensity was quantified with ImageJ software.

### Immunoprecipitation assay

The Pierce™ Classic Magnetic Bead-Based IP/Co-IP Kit (Invitrogen; cat. #88804) was used as recommended by the manufacturer. Following preparation as described in the western blot section, lysates (500-1000 µg) were immunoprecipitated with 2-4 µg of primary antibody or species-matched IgG control overnight at 4 °C. Following the addition of 30 μL Protein A/G beads, immune complexes were incubated for 2 hours at 4 °C. The beads were then magnetically captured, washed three times with ice-cold IP Lysis/Wash Buffer and once with ultrapure water, and finally analyzed by western blotting or mass spectrometrysamples were then analyzed via western blotting or mass spectrometry.

### Subcellular Fractionation and Western Blot Analysis

The Minute™ Cytoplasmic and Nuclear Extraction Kit (Invent Biotechnologies; cat. #SC-003) was used as recommended by the manufacturer. Cells were grown to 90-100% confluency in culture dishes. After removing the medium, cells were washed twice with ice-cold PBS. Cytoplasmic extraction buffer was added to cover the cell monolayer, followed by incubation on ice for 15 minutes. Cells were then scraped and lysates were transferred to1.5 mL tubes, vigorously vortexed for 15 seconds, and centrifuged at maximum speed in a microcentrifuge for 5 minutes at 4 °C. The supernatant (cytoplasmic fraction) was collected into a newtube. The pellet was washed once with 0.5 mL ice cold PBS to reduce cytoplasmic contamination, then resuspended in nuclear extraction buffer, vortexed vigorously for 15 seconds, and incubated on ice for 3 minutes. The vortex and incubation step were repeated four additional times (15 seconds vortex followed by 1 min on ice). The nuclear extract was clarified by centrifugation at 14,000-15,000 × g for 30 seconds using a filter tube assembly. Proteins from both fractions were either analyzed immediately by western blot or stored at -80 °C.

### Chromatin immunoprecipitation assay

Chromatin immunoprecipitation was performed with the Simple ChIP Plus Sonication Chromatin IP Kit (Cell Signaling Technology; cat. #56383). Cells were processed following the manufacturer's guidelines, and chromatin was fragmented via ultrasonication. Lysates were incubated overnight at 4 °C with the specified primary antibody or an appropriate IgG isotype control. Following cross-link reversal, the extracted DNA was quantified and used as template for qPCR with primers encompassing the KIF26A promoter region. The primer sequences are as follows: Forward: GAGGTGACTCCAGCCTTGTG; Reward: CGCAACTTCAGCGCTTCTAC.

### Single-cell gel electrophoresis assays

DNA double-strand breaks (DSBs) were assessed using the Comet Assay DNA Kit (Abcam; cat. #KTA3040). Following treatment, 5 × 10^5^ cells were prepared for each experiment. The assay was conducted following the manufacturer's guidelines. Samples were analyzed microscopically using Cytation5 (BioTek), and tail moments were quantified with CASP software.

### Laser micro-irradiation and live-cell imaging

Experimental procedures followed previously reported methodologies 12. Briefly, HCT116 and RKO cells were transduced with EGFP-KIF26A (GeneChem, Shanghai, China) and cultured in glass-bottom dishes (Cellvis; cat. #D35-20-1-N). Cells were incubated in medium containing 5 mM BrdU (Beyotime; cat. #ST1056) for 24 hours, then stained with Hoechst 33342 (Beyotime; cat. #C1056) for 30 minutes. After rinsing, microirradiation was performed with a 405-nm laser (Nikon A1R microscope, Tokyo, Japan) under NIS-Elements software control. Images were acquired every 40 seconds for 5 minutes and exported in TIFF format.

### Immunohistochemistry

Tumor tissues were fixed in 4% paraformaldehyde, embedded in paraffin, and sectioned at 5 μm. Following deparaffinization and rehydration, antigen retrieval was performed in ethylenediaminetetraacetic acid buffer (pH 9.0) using microwave heating. After blocking endogenous peroxidase, sections were incubated overnight at 4 ºC with primary antibodies. Detection was carried out using an ABC Kit (Pierce; cat. #32020) with DAB as the chromogen, followed by hematoxylin counterstaining, dehydration through graded ethanol and xylene, and mounting with neutral balsam.

### Luciferase reporter assays

HCT116 and RKO cells were seeded into 48-well plates. We introduced PRL-TK and pGL3-KIF26A-luc plasmids into cells using Lipofectamine 2000 (Invitrogen; cat. #11668019). The Dual-Luciferase Reporter Assay System (Promega; cat. #E1910) was employed to measure both Firefly and Renilla luciferase activities after 48 hours. Cells were rinsed with PBS, lysed with 65 µL of 1× PLB on a shaker at 200 rpm for 2 hours. Following centrifugation at 1000 g for 5 minutes, 20 µL aliquots of the supernatants were transferred to a white 96-well plate, and luminescence was measured with Cytation5 (BioTek). Firefly luciferase signals were normalized to PRL-TK.

### Homologous recombination (HR) and NHEJ reporter assays

HR and NHEJ reporter plasmids (pDR-GFP and EJ5-GFP, respectively) and the I-SceI expression plasmid were procured from GeneChem (Shanghai, China). We seeded HCT116 and RKO cells in 6-well plates and introduced reporter plasmids using Lipofectamine 2000 (Invitrogen; cat. #11668019). Puromycin (Beyotime; cat. #ST-551) was employed to enrich transfected cells. Cells were then transfected with the I-SceI plasmid to induce double-strand breaks. After 48 hours, cells were collected for flow cytometry. HR or NHEJ repair events were indicated by green fluorescence. Flow cytometry was conducted using a FACSAria (BD Biosciences), and data analysis was performed with FlowJo software.

### Tumor growth experiments in mice

Animal experiments were conducted according to the Institutional Animal Care and Use Committee of the Chinese Academy of Medical Sciences. Procedures were approved by the Animal Care Committee of the Chinese Academy of Medical Sciences (NCC2022A061). Five-week-old NSIG (strain #14006A) male mice were purchased from BEIJING HFK BIOSCIENCE CO., LTD. Mice were kept under pathogen-free conditions (22 °C, 60% humidity) with a standardized 14-hour light/10-hour dark photoperiod.

Subcutaneous tumors were established by injecting 4 × 10^6^ cancer cells resuspended in 100 μL PBS and 50 μL Matrigel (Corning; cat. #354234) into mice. CRT-treated mice received X-irradiation (4 Gy per session, MultiRad225, Faxitron) and intraperitoneal Oxaliplatin (5 mg/kg) on alternating days for four treatments. Mice in the histone deacetylase inhibitors (HDACi) group received Vorinostat (5 mg/kg) intraperitoneally on alternate days for three treatments. Tumor growth was monitored every 2-3 days with digital calipers. Tumor volume was calculated as: volume = length × width² ÷ 2. When tumor volume hit 2000 mm³ or upon reaching the study's conclusion at 30 days, mice were euthanized.

To evaluate treatment efficacy, we determined the tumor growth inhibition rate (TGI%) to quantify changes in tumor volume (TV) across various treatment regimens. The TGI% was computed using the following approach:



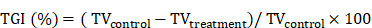



where
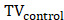
is the TV for the control animals at the endpoint, 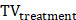
is the TV of the treated group at the endpoint time.

### Bioinformatics analysis

Drug activity data and KIF26A mRNA expression levels were obtained from the GDSC database via the CellMiner Cross Database (CellMiner CDB) platform. Forty-eight drugs with a Spearman correlation index > 0.2 or < -0.2 and *P* < 0.05 were identified. From this set, 22 drugs annotated in the GDSC database were selected to construct a bar plot.

To examine the relationship between KIF26A expression and cancer cell responsiveness to radiotherapy and chemotherapy, DNA repair-related gene expression datasets were obtained from GEO (accession numbers GSE60331 and GSE119409) and from the National Genomic Data Center (Project ID: PRJCA027384).

Immunohistochemically stained images of human cancer tissue samples were acquired from the Human Protein Atlas (https:// www.proteinatlas.org).

Expression levels of KIF26A in 50 paired colorectal cancer and adjacent normal tissues were obtained from The Cancer Genome Atlas (TCGA) (https://www.cancer.gov/ccg/research/genome-sequencing/tcga).

Pan-cancer KIF26A expression data across tumor and normal tissues were downloaded from the UCSC Xena platform (https://xena.ucsc.edu/).

### Statistical analysis

R software (version 4.5.0) and GraphPad Prism (version 9.5.1) were employed for statistical analyses. When covariate adjustment was not required, correlations between categorical variables were evaluated with Fisher's exact test, and the Wilcoxon rank-sum test was applied for comparisons between continuous and binary variables. Otherwise, F-tests were used to compare generalized linear models, one including predictor variables. Associations between continuous variables were measured using Spearman's rank correlation. Functional assay results were depicted as mean ± SEM. Group differences were analyzed using Student's *t*-test. Multivariate survival analyses were performed using Cox proportional hazards models, and univariate survival analysis was performed using the log-rank test, with Kaplan-Meier curves generated to visualize survival outcomes. All statistical analyses were two-sided, employing a significance threshold of *P* < 0.05.

## Results

### KIF26A functions as a DNA damage response protein

**Figure 1:**
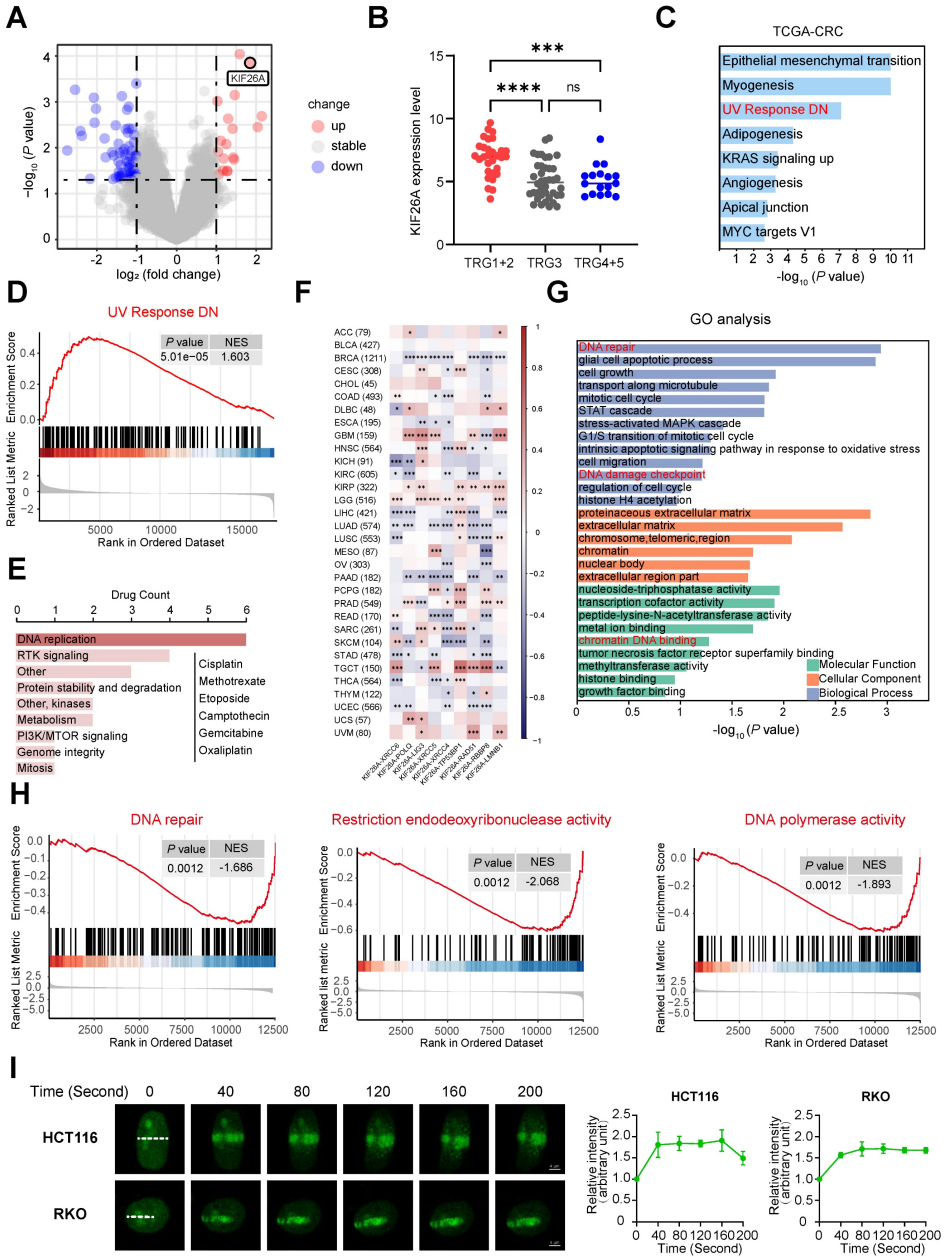
** KIF26A functions as a DNA damage response protein. (A)** DNA array analysis in responders and non-responders. 132 DEGs (*P* < 0.05 and Fold Change ≥ 2.0 or ≤ 0.5) were found. **(B)** KIF26A expression level among samples of responders (TRG1+2)(n = 30), intermediate responders (TRG3)(n = 37) and non-responders (TRG4+5)(n = 14). Data are shown as mean±SD. ***P < 0.001; ****P < 0.0001; ns, not significant, as determined by one-way ANOVA. **(C)** Gene Set Enrichment Analysis (GSEA) of the differentially expressed genes between the high and low KIF26A expression groups (divide into high and low groups based on the median) from TCGA-COADREAD dataset. **(D)** The results of Gene Set Enrichment Analysis (GSEA) focusing on the UV Response DN pathway. **(E)** Drug activity data and mRNA expression level of KIF26A in colon or CRC cell lines were downloaded from CellMinerCDB. Spearman correlation coefficient between drug activity and KIF26A expression level was calculated and the correlation was tested. The bar plot shows the number of target pathways of the 22 filtered drugs with GDSC annotation. **(F)** KIF26A expression is compared to that of genes coding for DNA repair factors. Unsupervised hierarchical clustering of the Pearson's correlation coefficients (PCCs) of gene expression analyses with studied genes depicted on the x axis across TCGA studies. Cancer acronyms are on the y axis; nominally significant PCCs are indicated by *P < 0.05, **P < 0.01 or ***P < 0.001. The TCGA cancer acronyms are listed in the Abbreviations section. **(G)** Gene Ontology Enrichment Analysis (GO) of differentially expressed genes in cells with KIF26A overexpression (OE) or control. **(H)** The results of Gene Set Enrichment Analysis (GSEA) focusing on the DNA repair related pathways of the differentially expressed genes between KIF26A-overexpressing or control. **(I)** Live-cell imaging of EGFP-KIF26A proteins recruitment to laser-induced DNA damage sites in HCT116 and RKO cells. Confocal images are presented, with white dashed lines highlighting irradiated areas. The accumulation of EGFP-KIF26A at these sites was measured (n = 3). Scale bars, 4 µm. D, H and I, NES, normalized enrichment score. Error bars, mean ± SD. I, three independent biological replicates were performed.

In our previous study, we performed gene expression analysis on 81 rectal cancer patients who received preoperative CRT and showed varied responses 10. Compared to the resistant group, the CRT-sensitive group exhibited markedly higher KIF26A expression levels (**Figure 1A, B**). We explored how KIF26A affects patient sensitivity to chemoradiotherapy. Colorectal cancer data from TCGA were stratified based on KIF26A expression levels. GSEA indicated a significant association between KIF26A abundance and UV_RESPONSE_DN pathway enrichment (**Figure 1C, D**). Because radiotherapy and certain chemotherapeutic agents act primarily by inducing DNA damage, we analyzed the CellMiner Cross-Database (CellMiner CDB). The findings showed that KIF26A expression correlates with sensitivity to DNA-damaging agents such as Oxaliplatin, Cisplatin, and Methotrexate (**Figure 1E**). TCGA pan-cancer analysis also uncovered a notable association between KIF26A and DNA repair genes across multiple cancers, including colorectal cancer (**Figure 1F**). Therefore, we speculated that KIF26A may affect sensitivity to radiotherapy and chemotherapy by modulating DNA repair. We generated stable KIF26A knockdown and overexpression in HCT116 and RKO colorectal cancer cells, with efficiency confirmed by qPCR and western blot analysis (Supplementary Figure S1A, B). RNA-seq analysis of KIF26A-overexpressing RKO cells (Supplementary Table S5) showed 253 downregulated and 78 upregulated genes compared with vector controls (*P*_adjust < 0.05, fold change < 0.5 or > 2) (Supplementary Figure S1C, D). GO and GSEA analysis showed enrichment in DNA repair, cell growth, histone acetylation, cell adhesion, and PI3K-AKT signaling pathways (**Figure 1G,** Supplementary Figure S1E). Pathways associated with DNA damage repair, including DNA repair, restriction endonuclease activity, and DNA polymerase activity, were suppressed in KIF26A-overexpressing cells (**Figure 1H**). To identify interaction partners of KIF26A, we performed immunoprecipitation in HCT116 cells followed by mass spectrometry. KIF26A was found to interact with at least 75 proteins, including DNA repair-related proteins such as Ku70, Ku80, XRCC1, RPA, and PARP1 (Supplementary Table S6). To test whether KIF26A functions in the DNA damage response, EGFP-KIF26A was expressed in HCT116 and RKO cells. Upon laser-induced DNA damage, KIF26A rapidly accumulated at damage sites (**Figure 1I**). This rapid recruitment suggests that KIF26A may function as an early responder and active participant in the DNA damage repair machinery. Collectively, these findings point to the involvement of KIF26A in DNA damage repair.

### KIF26A inhibits DNA damage repair

**Figure 2:**
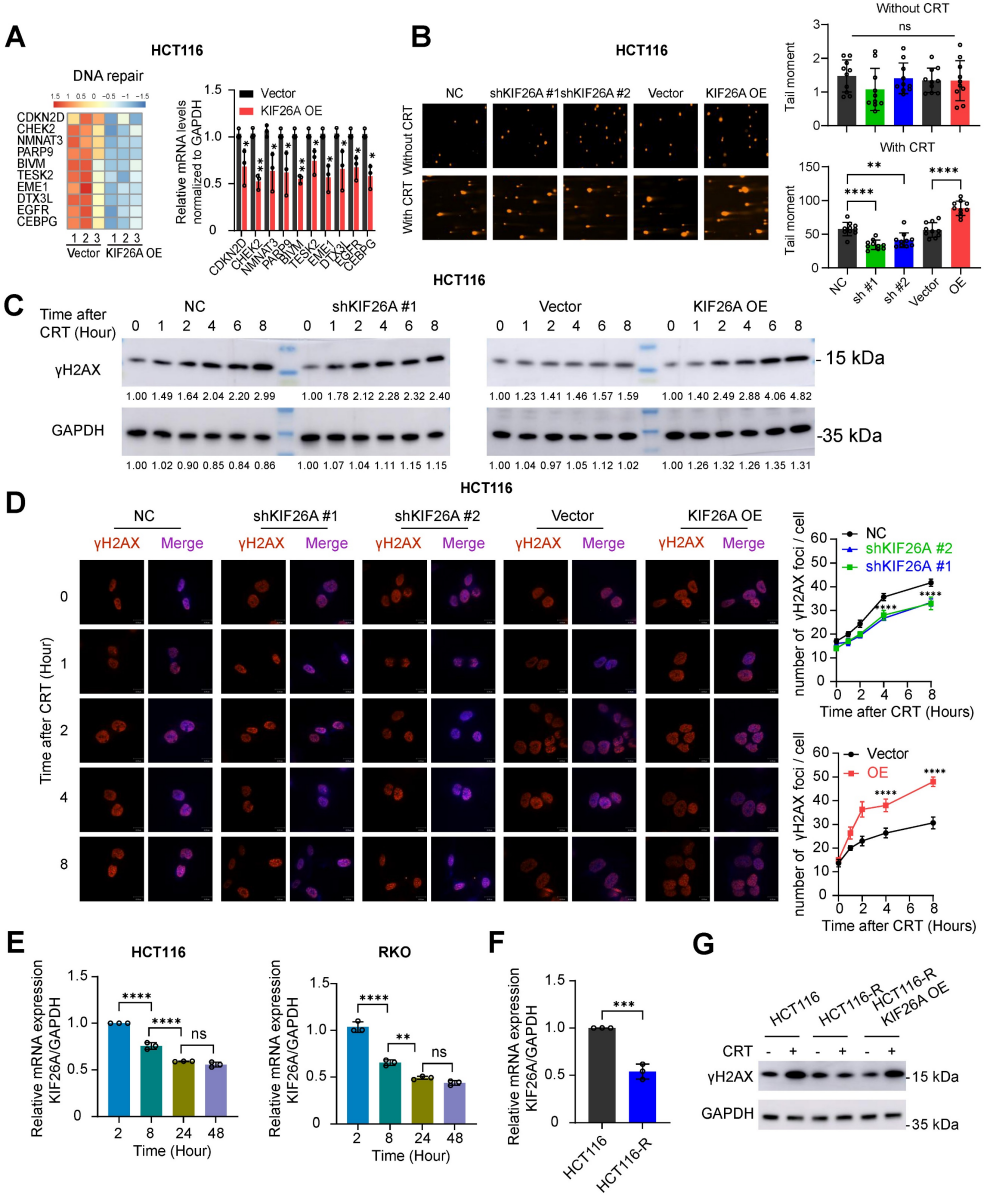
** KIF26A inhibits DNA damage repair. (A)** The heatmap illustrates the top 10 genes in the DNA repair pathway significantly enriched by GSEA (left). Verification of the expression changes of the differentially expressed genes in the DNA repair pathway in HCT116 cell (right)(n = 3). **(B)** DNA damage in KIF26A-modulated HCT116cells after CRT treatment was assayed by comet assay (n = 10). Scale bars, 100 µm. **(C)** C, D. DNA double-strand breaks expressed by γ-H2AX level in KIF26A knockdown or overexpressing HCT116 cells treated with or without CRT. (C) Shows γ-H2AX by western blot analysis. **(D)** Shows images of γ-H2AX foci in cells at various time points of IR as indicated (n = 3). Scale bars, 10 µm. **(E)** Expression changes of KIF26A with increasing CRT treatment duration in HCT116 and RKO cells (n = 3). **(F)** Expression of KIF26A in HCT116 parental cells (HCT116) and HCT116 chemoradiotherapy-resistant cells (HCT116-R) (n = 3). **(G)** Western blot analyses of the levels of γH2AX in the indicated cells treated with or without CRT. A, C and G, CRT group treated with radiation (8 Gy) and chemotherapy (2 μg/mL of Oxaliplatin). E, treatment by radiation (4 Gy) and chemotherapy (1 μg/mL of Oxaliplatin).A, B and D-F, *P < 0.05, **P < 0.01, ***P < 0.001 or ****P < 0.0001; ns, not significant; A, D and F, determined by Student's *t*-test; B and E, determined by one-way ANOVA; *P* value of less than 0.05 indicates a statistical difference. Error bars, mean ± SD. For all panels, three biological independent replicates were performed.

Expression of DNA repair-related genes was assessed through RT-qPCR and found to be significantly downregulated in KIF26A-overexpressing cells (**Figure 2A**, Supplementary Figure S2A). The neutral comet assay showed that at 18 hours post-CRT treatment, tail moments were shorter in KIF26A-sh cells compared with negative controls, whereas KIF26A overexpression prolonged tail moments, indicating impaired repair (**Figure 2B**, Supplementary Figure S2B). Increased γ-H2AX levels detected through Western blot and immunofluorescence confirmed that KIF26A overexpression increased CRT-induced DNA damage, while KIF26A knockdown reduced γ-H2AX in treated cells (**Figure 2C, D,** Supplementary Figure. S2C, D). We examined KIF26A expression after chemoradiotherapy and found that it declined progressively with longer treatment durations (**Figure 2E**), suggesting a role in secondary resistance. A chemoradiotherapy-resistant HCT116 cell line was established and validated by limiting dilution, IC_50_, CCK-8, and colony formation assays (Supplementary Figure. S2E-H). KIF26A expression was decreased in resistant HCT116 cells (**Figure 2F**). We overexpressed KIF26A in the HCT116-R cells (Supplementary Figure. S2I). After chemoradiotherapy treatment, HCT116-R cells showed minimal DNA damage; however, KIF26A overexpression abolished this resistance (**Figure 2G**). Taken together, the evidence from these experiments suggests that KIF26A inhibits DNA damage repair and that loss of KIF26A contributes to both primary and secondary chemoradio-resistance.

### KIF26A inhibits the NHEJ repair pathway by binding to Ku70

**Figure 3:**
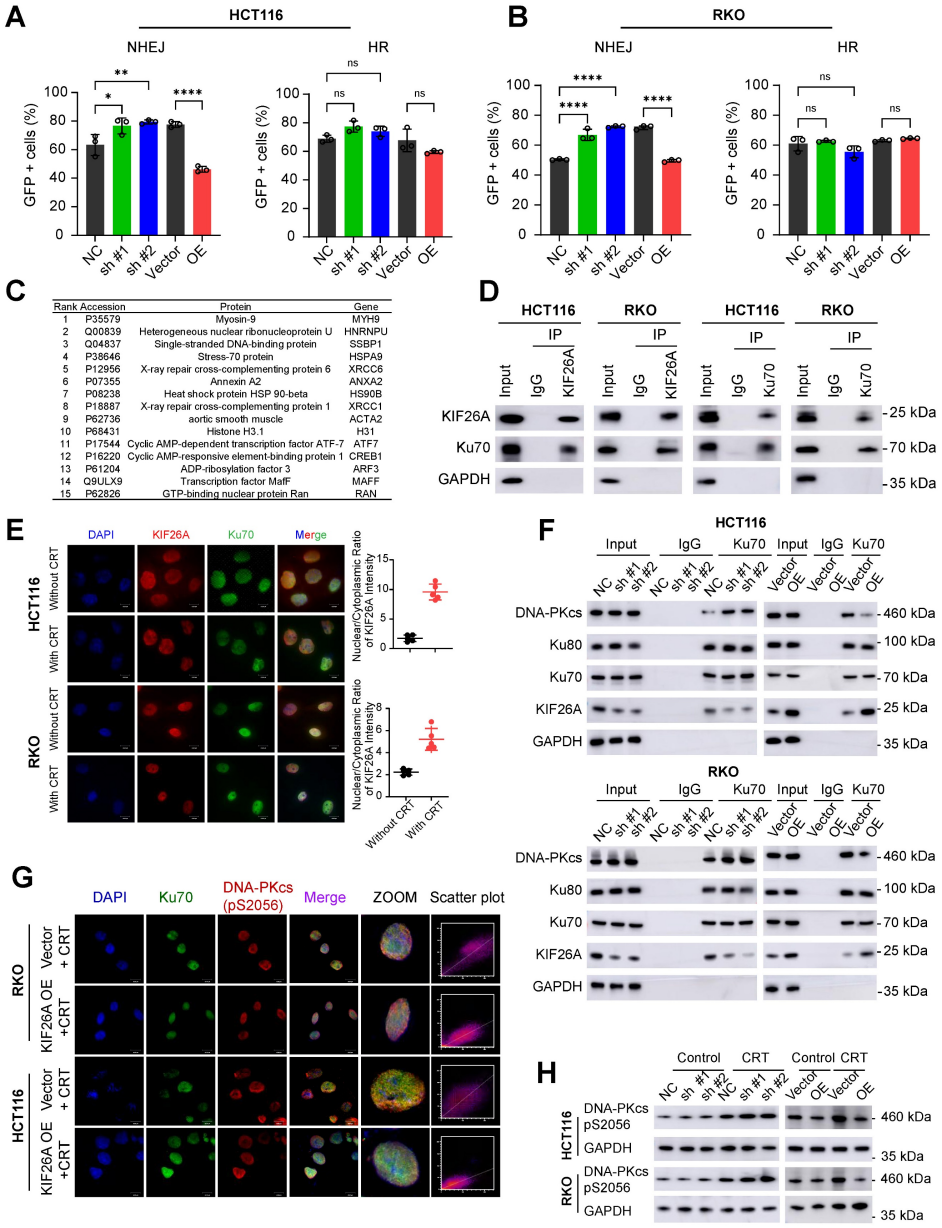
** KIF26A inhibits the NHEJ repair pathway by binding to Ku70. (A, B)** In HCT116 (A) and RKO (B) cells characterization of DSB repair pathways by pEJ5-GFP (left) and DR-GFP (right). GFP+ cell percentages were quantified via FACS (n = 3). **(C)** Mass spectrometry identified 15 top proteins associated with KIF26A in HCT116 cells overexpressing KIF26A. Cell lysates underwent KIF26A-antibody immunoprecipitation, with IgG-antibody as a negative control. **(D)** In HCT116 and RKO cells, Western blot probed KIF26A and Ku70 proteins immunoprecipitated via anti-KIF26Aor anti-Ku70antibodies, with IgG as the control. **(E)** Immunofluorescence analysis of KIF26A and Ku70 in HCT116 and RKO cells. DAPI labels nuclei. Scale bars: 10 µm. Shows the colocalization of KIF26A and Ku70 before and after CRT treatment (n = 5). **(F)** IP-WB analysis of Ku70-Ku80-DNA-PKcs complex with anti-Ku70 antibody in HCT116 (upper) and RKO (lower) cells. **(G)** Confocal images of DNA-PKcs (Ser2056, red) and Ku70 (green) in KIF26A-overexpressing or control cells after CRT treatment. Last column: Cell pixel intensity scatter plot. The last column shows the scatter plot of red and green pixel intensities in different treatment groups. **(H)** Western blot analysis of DNA-PKcs phosphorylation in HCT116 and RKO cells under different treatments. E, G and H, CRT group treated with radiation (8 Gy) and chemotherapy (2 μg/mL of Oxaliplatin). A, ****P < 0.0001 or ns, not significant; determined one-way ANOVA; *P* value of less than 0.05 indicates a statistical difference. Error bars, mean ± SD. For all panels, three biological independent replicates were performed.

NHEJ repairs DNA DSBs by directly ligating DNA ends without requiring a template 15. NHEJ is primarily active during the G1 and G2/M phases of the cell cycle and is as the predominant repair pathway in mammalian cells 16. Homologous recombination (HR) repairs DSBs using homologous sequences 15, mainly during the S and G2 phases 17. To investigate which pathway KIF26A affects, we used pEJ5-GFP and DR-GFP reporters to quantify NHEJ and HR activity. The NHEJ reporter assay showed that KIF26A overexpression decreased NHEJ efficiency after DSB induction, whereas KIF26A knockdown had the opposite effect. In contrast, KIF26A expression had a limited impact on HR activity (**Figure 3A, B**, Supplementary Figure S3A, B). Mass spectrometry data showed that X-ray repair cross-complementing protein 6 (XRCC6), commonly known as Ku70, a crucial NHEJ factor forming the Ku70/Ku80 heterodimer, interacts with KIF26A (**Figure 3C**). Reciprocal co-immunoprecipitation (CoIP) using antibodies against KIF26A or Ku70 confirmed this association in colorectal cancer cells (**Figure 3D**). To directly assess the dynamics of the KIF26A-Ku70 interaction, we quantified their binding affinity by CoIP before and after CRT. The assays revealed a significant increase in the KIF26A-Ku70 association post-treatment (Supplementary Figure S3D). Immunofluorescence staining showed KIF26A localized to both cytoplasm and nucleus, while Ku70 was predominantly nuclear. KIF26A and Ku70 displayed a degree of spatiotemporal overlap in their distribution. Notably, CRT treatment induced a substantial shift in KIF26A distribution towards the nucleus, implying that therapy promotes its nuclear accumulation, which may facilitate its role in the DNA damage response (**Figure 3E** and Supplementary Figure S3E). Their colocalization is evident in specific subcellular regions, suggesting potential interactions or coordinated functions within the cellular environment. To identify the specific regions of KIF26A that mediate its interaction with Ku70, we first analyzed the protein domain architecture of KIF26A using the NCBI Conserved Domains. Based on this analysis, we designed and generated a series of FLAG-tagged putative functional domains: aa 1-830 (N-terminal region), aa 850-1240, and aa 1330-1728 (C-terminal region) (Supplementary Figure. S3B). We also generated a Myc-tagged Ku70. These constructs were then overexpressed in HCT116 cells. IP-WB analyses showed that the C-terminal region (aa 1330-1728) mediated binding of KIF26A to Ku70 (Supplementary Figure. S3C). When a DNA DSB occurs, the Ku70/Ku80 heterodimer quickly attaches to the break ends and recruits activated DNA-PKcs. Activated DNA-PKcs then recruits other NHEJ proteins such as Artemis, XRCC4, and DNA ligase IV. These proteins process and connect DNA ends, ultimately repairing the DNA 18-20. These steps constitute the NHEJ repair pathway that efficiently fixes DSBs and stabilizes the genome. We hypothesized that the binding of KIF26A to Ku70 might disrupt Ku70's interaction between with Ku80 and DNA-PKcs, thereby affecting the initiation of the NHEJ repair pathway. We used CoIP-WB to examine whether KIF26A inhibits the formation of the Ku70-Ku80-DNA-PKcs complex and found that increased KIF26A expression negatively affected DNA-PKcs recruitment, whereas downregulation of KIF26A enhanced it (**Figure 3F**). Immunofluorescence staining was performed on DNA-PKcs (Ser2056) and Ku70 cells. After CRT treatment, the colocalization coefficient between DNA-PKcs (Ser2056) and Ku70 was reduced in KIF26A-overexpressing cells, indicating that KIF26A prevented the recruitment of DNA-PKcs to Ku70 (**Figure 3G**). We assessed DNA-PKcs activation by analyzing phosphorylation using western blotting. In KIF26A-overexpressing cells, reduced phosphorylation of DNA-PKcs at serine 2056 was observed, confirming KIF26A's role in modulating DNA-PKcs activation. Conversely, the downregulation of KIF26A expression increased DNA-PKcs phosphorylation at serine 2056 (**Figure 3H**). To directly test whether DNA-PKcs is the functional downstream effector, we treated KIF26A-knockdown cells with the DNA-PKcs inhibitor NU7441 21. NU7441 rescued the CRT resistance induced by KIF26A knockdown, confirming DNA-PKcs as the critical functional target within this pathway (Supplementary Figure S3F). In summary, these findings indicate that KIF26A disrupts the Ku70/80-DNA-PKcs interaction by competing with DNA-PKcs for direct binding to Ku70.

### KIF26A increases chemoradiotherapy sensitivity by suppressing DNA repair

**Figure 4:**
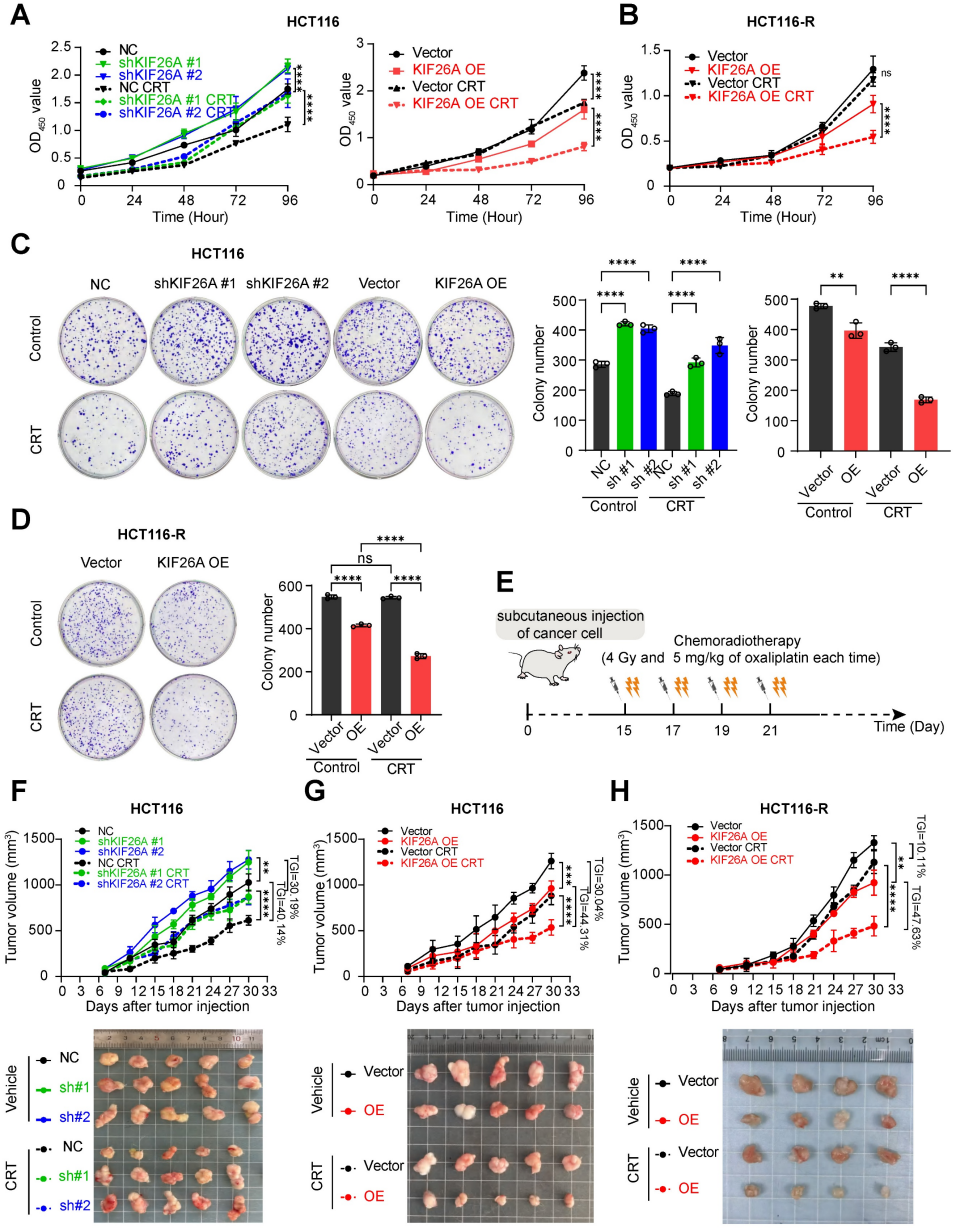
** KIF26A increases chemoradiotherapy sensitivity by suppressing DNA repair. (A)** CCK8 assay showed the proliferation ability of HCT116 cells following KIF26A knockdown or overexpression (n = 6). **(B)** CCK8 assay showed the proliferation ability of HCT116-R cells following KIF26A overexpression (n = 6). **(C)** The colony formation assay showed the proliferation ability of HCT116 cells following KIF26A knockdown or overexpression (n = 3). **(D)** The colony formation assay showed the proliferation ability of HCT116-R cells following KIF26A overexpression (n = 3). **(E)** Cancer cells (HCT116, HCT116-R, RKO or H520) were subcutaneously injected into NSG mice and then treated with radiation (4 Gy) and Oxaliplatin (5 mg/kg) or not at the indicated time points (n = 4-5 per group). **(F, G, H)** Tumor growth curves (upper) and excised tumor images (lower) of control and KIF26A-knockdown tumors (F), KIF26A-overexpression tumors (G) in HCT116 cells and KIF26A-overexpression tumors in HCT116-R (H) cells with or without CRT treatment (n = 4-5 per group). TGI, percentageof tumor growth inhibition. A-D, CRT group treated with radiation (4 Gy) and chemotherapy (1 μg/mL of Oxaliplatin); Control group treated with the same volume of DMSO as the CRT group and without radiation. A-D and F-H, **P < 0.01, ***P < 0.001, ****P < 0.0001 or ns, not significant; determined one-way ANOVA; *P* value of less than 0.05 indicates a statistical difference. Error bars, mean ± SD. A-D, three biological independent replicates were performed.

Chemoradiotherapy induces DNA damage via multiple pathways, often resulting in DSBs, which lead to cell death or senescence. Resistance may develop through mechanisms that reduce DSB formation or enhance repair 22, 23. To test whether KIF26A modulates sensitivity to IR and chemotherapy, cells were treated with radiotherapy or Oxaliplatin. Reduced KIF26A expression lessened the cells' sensitivity to Oxaliplatin and radiotherapy relative to the control group, while increased KIF26A expression heightened the cells' chemosensitivity and radiotherapy response (Supplementary Figure S4A-D). The cells were then subjected to combined chemoradiotherapy to evaluate synergistic effects on cell proliferation and colony-forming abilities. Following chemoradiation treatment, cells with KIF26A knockdown exhibited significantly greater proliferation and colony-forming abilities compared to control cells, whereas these abilities were notably reduced when KIF26A was overexpressed (**Figure 4A, C**, Supplementary Figure S5A, B). In chemoradiotherapy resistant cell, KIF26A overexpression enhanced the chemo-radiosensitivity of the HCT116-R cell (**Figure 4B, D**). Similarly, the growth of subcutaneous tumors increased in the shKIF26A groups, and the transplanted tumors showed better chemoradiotherapy responses in KIF26A overexpression groups (**Figure 4E-G**, Supplementary Figure S5C, D). *In vivo* studies also showed that KIF26A overexpression increased the sensitivity of chemoradiotherapy-resistant cells. (**Figure 4H**, Supplementary Figure S5E). To substantiate the mechanistic basis of tumor growth inhibition *in vivo*, immunohistochemical analysis was performed on HCT116 xenografts. The results demonstrated that the combination treatment markedly increased levels of DNA damage (γ-H2AX) and apoptosis (Cleaved Caspase-3). Although total KU70 protein levels were not markedly altered, the profound persistence of γ-H2AX foci suggests a functional impairment of the NHEJ repair pathway. Concurrently, cellular proliferation (Ki67) was also reduced (Supplementary Figure S6A). Furthermore, we conducted identical validation experiments in the lung cancer cell line H520 and obtained consistent results (Supplementary Figure S7A-C). Treatment toxicity was evaluated by monitoring the body weight of mice (Supplementary Figure S4E). These results demonstrate that KIF26A enhances cellular sensitivity to radiotherapy and chemotherapy.

### Dynamic regulation of KIF26A transcription by HDAC/HAT-mediated promoter acetylation

**Figure 5:**
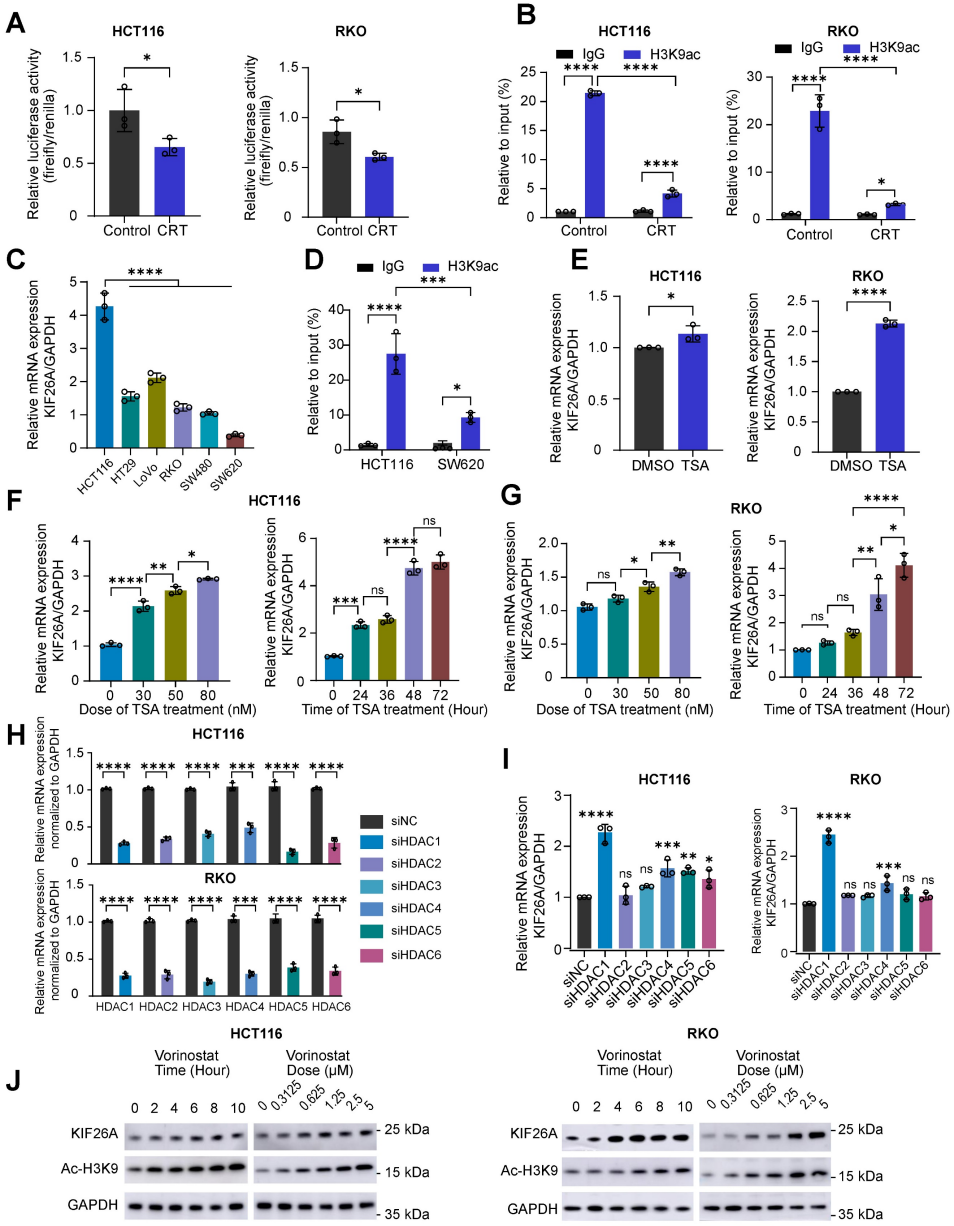
** Dynamic regulation of KIF26A transcription by HDAC/HAT - mediated promoter acetylation. (A)** Dual-luciferase reporter assays were performed to evaluate the promoter activity of the KIF26A in HCT116 and RKO cells before and after CRT treatment (n = 3). **(B)** ChIP assay assessed KIF26A promoter acetylation in HCT116 and RKO cells before and after CRT treatment (n = 3). **(C)** KIF26A expression in different parental cell lines were quantified by qPCR (n = 3). **(D)** ChIP assay assessed KIF26A promoter acetylation in HCT116 and SW620 cells (n = 3). **(E)** Expression levels of KIF26A in HCT116 and RKO cells with or without TSA treatment. TSA group treated with 40 nM TSA; Control group treated with the same volume of DMSO as the TSA group (n = 3). **(F)** The relationship between KIF26A expression levels and the concentration (left) or duration (right) of TSA treatment in HCT116 cells (n = 3). **(G)** The relationship between KIF26A expression levels and the concentration (left) or duration (right) of TSA treatment in RKO cells (n = 3). **(H)** After individual knockdown of HDAC1-6 in HCT116 and RKO cells, the efficiency was validated by qPCR (n = 3). **(I)** In HCT116 and RKO cells, KIF26A expression following individual knockdown of different HDAC family members (n = 3). **(J)** Western blot assessed KIF26A expression changes with Vorinostat treatment duration or dose in HCT116 and RKO cells. A and B, CRT group treated with radiation (4 Gy) and chemotherapy (1 μg/mL of Oxaliplatin); Control group treated with the same volume of DMSO as the CRT group and without radiation. A-I, *P < 0.05, **P < 0.01, ***P < 0.001 or ****P < 0.0001; ns, not significant; A, E and H, determined by Student's *t*-test; B-D, F, G and I, determined by one-way ANOVA; *P* value of less than 0.05 indicates a statistical difference. Error bars, mean ± SD. A-J, three biological independent replicates were performed.

To address chemoradiotherapy resistance, we investigated upstream factors influencing KIF26A expression. We first confirmed that CRT represses KIF26A promoter activity **(Figure 5A, B)**, aligning with our earlier observation of transcriptional inhibition **(Figure 2E)**. Since CRT is known to remodel the epigenome by altering histone acetylation 24-26, we performed chromatin immunoprecipitation assays and found it significantly reduces H3K9ac enrichment at the KIF26A promoter **(Figure 5B)**. Basal KIF26A expression and promoter acetylation were both higher in HCT116 than in SW620 cells **(Figure 5C, D)**, further supporting the role of acetylation in regulating KIF26A. Histone acetylation is regulated by HATs (adding acetyl groups) and HDACs (removing acetyl groups) 24, 25. We exposed the cells to TSA, a pan-HDACi, and observed that KIF26A expression rose significantly in a dose- and time-related manner (**Figure 5E**-**G**). Given that TSA has not yet been approved for clinical use, we decided to employ other analogous HDAC inhibitors which have been authorized for treating cancers in our subsequent studies. In HCT116 and RKO cells, individual siRNA-mediated depletion of HDAC1-6 deacetylases revealed that only knockdown of HDAC1 or HDAC4 provoked a marked rise in KIF26A transcript abundance, with HDAC1 silencing eliciting a more pronounced effect (**Figure 5H, I**). Subsequent investigations will primarily focus on Vorinostat, an orally bioavailable inhibitor that targets HDAC1-3, HDAC6-7, and HDAC11 histone deacetylases 27. This decision was further supported by Vorinostat's broader clinical application, with documented efficacy in colorectal 28, head and neck 26, lung 29, and breast cancers 30. We found that Vorinostat increased the protein level of KIF26A in a manner dependent on dose and time (**Figure 5J**). Taken together, our investigation showed that KIF26A expression is regulated by promoter acetylation, indicating that HDAC inhibitors are a viable strategy for its upregulation.

### HDACi in conjunction with chemoradiotherapy can sensitize cells that are resistant to chemoradiotherapy

**Figure 6:**
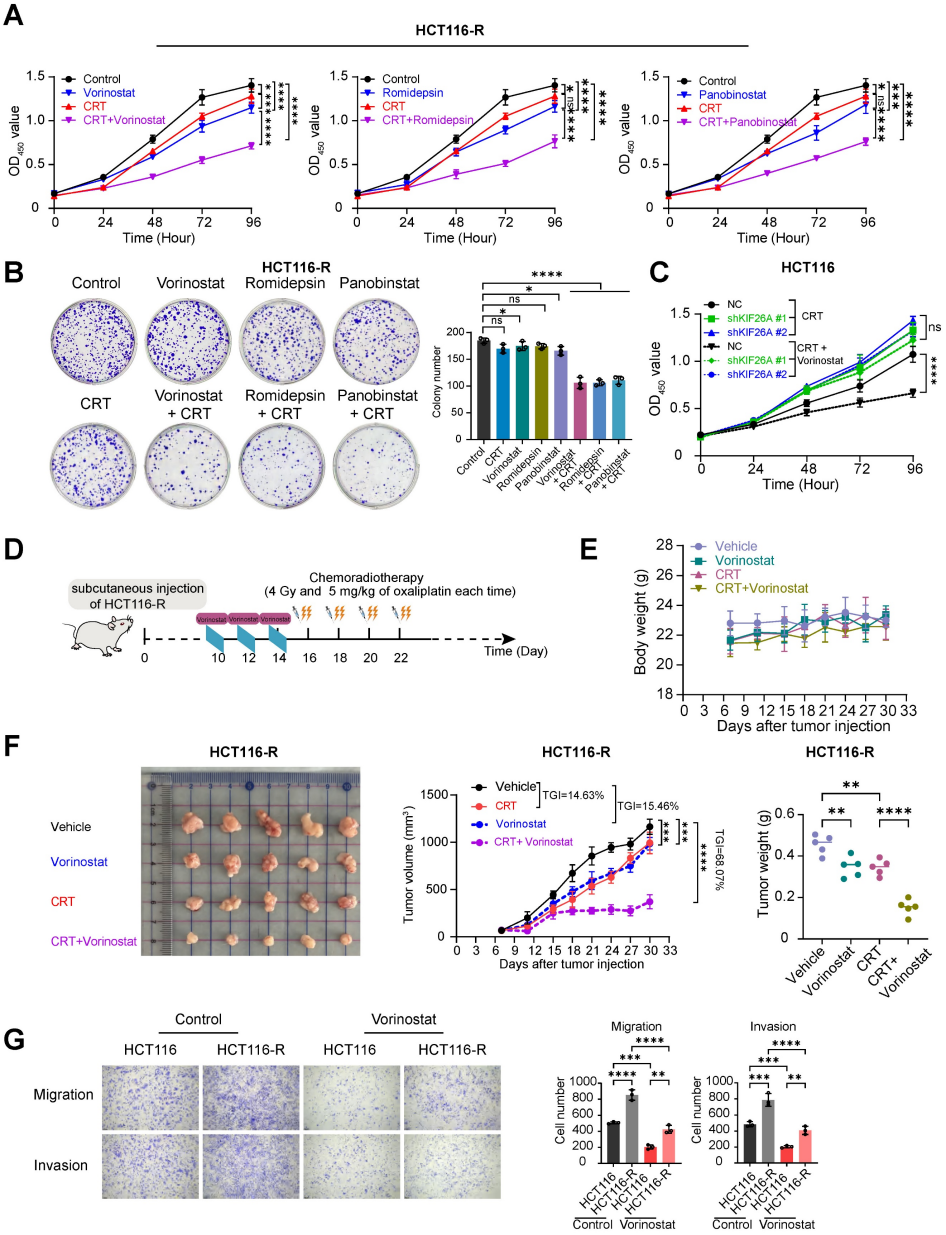
** HDACi in conjunction with chemoradiotherapy can sensitize cells that are resistant to chemoradiotherapy. (A)** CCK8 assay showed the proliferation ability of HCT116-R cells treated with or without CRT in the presence or absence of Vorinostat (left), Romidepsin (middle), and Panobinstat (right)(n = 6). **(B)** The colony formation assay showed the proliferation ability of HCT116-R cells treated with or without CRT in the presence or absence of Vorinostat, Romidepsin, and Panobinstat (n = 3). **(C)** CCK8 assay showed the proliferation ability of HCT116 control (NC) and KIF26A-knockdown (sh) cells following treatment with or without Vorinostat (n = 6). **(D)** HCT116-R cells were subcutaneously injected into NSG mice and then treated with radiation (4 Gy) and Oxaliplatin (5 mg/kg) or not, in the presence or absence of Vorinostat (5 mg/kg) at the indicated time points (n = 5 per group). **(E)** Body weight of control, CRT, Vorinostat and CRT+Vorinostat tumors (n = 5 per group). **(F)** Excised tumor images (Left), tumor growth curves (Middle) and tumor weight (Right) of control, CRT, Vorinostat and CRT+Vorinostat tumors (n = 5 per group). TGI, percentage of tumor growth inhibition. **(G)** The transwell assay showed the migration and invasion abilities of HCT116 and HCT116-R cells with or without the treatment of Vorinostat. Vorinostat group treated with 1 μM Vorinostat; Control group treated with the same volume of DMSO as the Vorinostat group. The treatment duration was 8 hours (n = 3).A and B, CRT group treated with radiation (4 Gy) and chemotherapy (2 μg/mL of Oxaliplatin); Control group treated with the same volume of DMSO as the CRT group and without radiation. Vorinostat (left), Romidepsin (middle), and Panobinstat (right) groups, the treatment concentrations of the three drugs are 1 μM, 5 nM and 5 nM. The treatment duration was 8 hours. Combination group cells were treated with each of the three HDAC inhibitors separately for 8 hours at the aforementioned concentrations followed by CRT treatment. C, CRT group treated with radiation (4 Gy) and chemotherapy (2 μg/mL of Oxaliplatin). Combination group cells were treated with Vorinostatfor 8 hours at the aforementioned concentrations followed by CRT treatment (1μM). A-C and E-G, *P < 0.05, **P < 0.01, ***P < 0.001 or ****P < 0.0001; ns, not significant; determined by one-way ANOVA; *P* value of less than 0.05 indicates a statistical difference. Error bars, mean ± SD. A-C and E-G, three biological independent replicates were performed.

Based on this mechanistic insight, we then explored the potential of HDAC inhibitors to improve therapeutic efficacy by increasing chemoradiotherapy sensitivity. For *in vitro* studies, Vorinostat was supplemented with two additional HDAC inhibitors: Romidepsin, which selectively inhibits HDAC1, 2, 4, and 6, and Panobinostat, a non-selective pan-HDAC inhibitor 31, 32. We treated cells with CRT alone, HDACi alone, or a combination of CRT and HDACi on the chemoradiotherapy-resistant cell line HCT116-R and found that while CRT and HDACi individually showed minimal effect on inhibiting cell proliferation, the pretreatment with HDACi followed by CRT demonstrated a marked suppression of cancer cell viability and growth (**Figure 6A, B**) These *in vitro* results indicate that HDACi may facilitate CRT-induced killing of HCT116-R cells. Subsequently, we treated KIF26A-knockdown cells with Vorinostat and CRT. We found that the chemo-radiosensitizing effect of HDACi was abolished in the knockdown group, indicating that KIF26A is an essential mediator of HDACi-induced sensitization to CRT (**Figure 6C**). Previous studies have shown that Vorinostat combined with radiotherapy improves outcomes in pediatric diffuse intrinsic pontine gliomas 33. *In vivo* mouse xenograft assays using HCT116-R cells confirm that Vorinostat significantly enhanced chemo-radiosensitivity (**Figure 6D-F**). Some studies have shown that radiotherapy can induce epithelial-mesenchymal transition (EMT) in some surviving cancer cells, endowing them with invasive capabilities and thereby facilitating cancer metastasis 34. We investigated HCT116-R cell lines and found enhanced migration and invasion abilities compared with the parental cell line. We explored whether Vorinostat could inhibit this metastatic phenomenon (**Figure 6G**). Results indicated that Vorinostat also inhibited distant metastasis caused by long-term low-dose chemoradiotherapy. Overall, Vorinostat has a significant therapeutic effect on chemoradiotherapy-resistant cell lines.

### Reduced KIF26A expression indicates poor prognosis for patients receiving chemoradiotherapy

**Figure 7:**
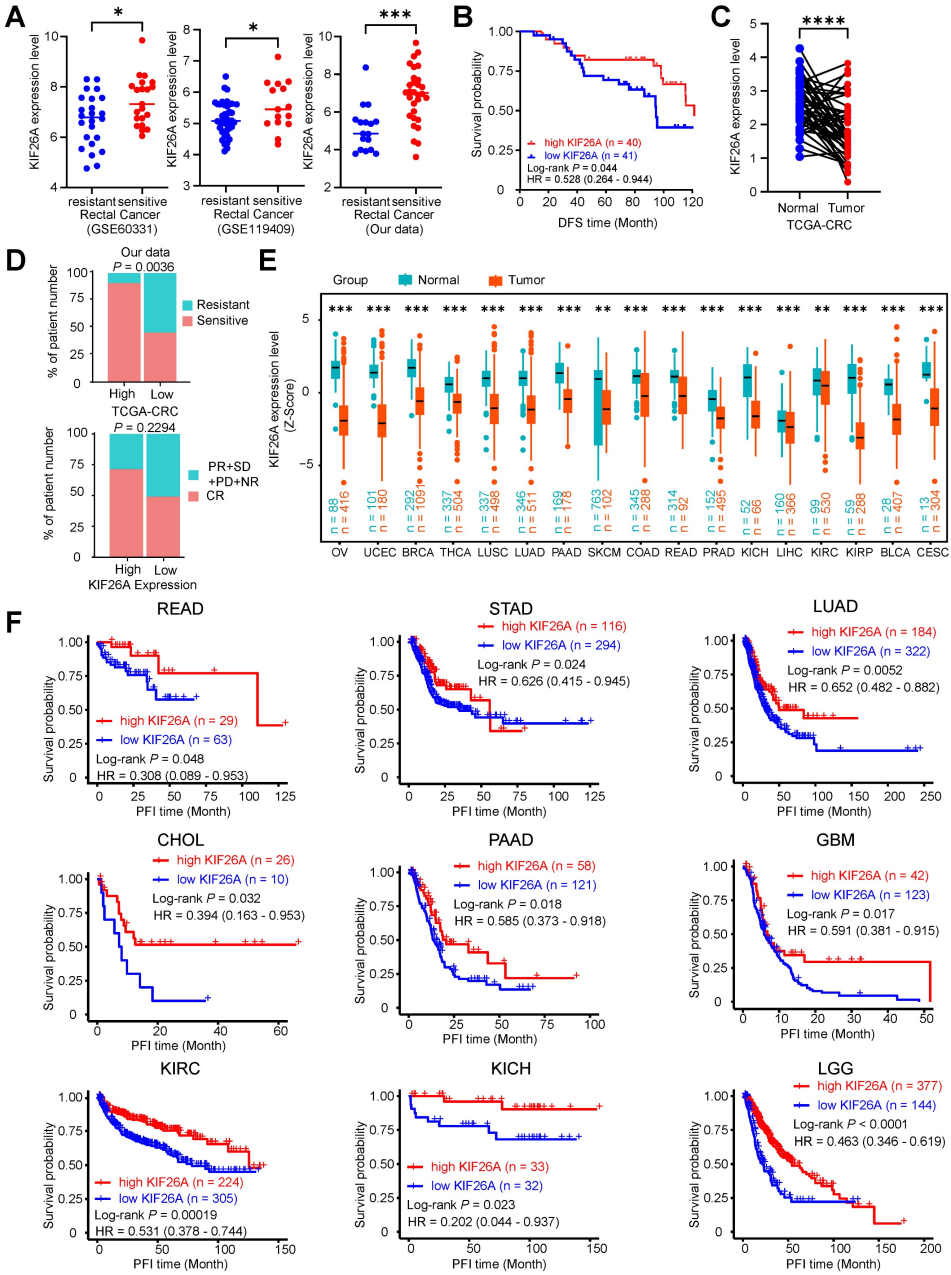
** Reduced KIF26A expression informs poor prognosis of patients to chemoradiotherapy. (A)** Validation was performed in several chemoradiotherapy-related GEO databases. Bar graphs (with scatter plots) show the expression levels of KIF26A in (from left to right): the GSE60331 cohort (Resistant, n = 24; Sensitive, n = 21), the GSE119409 cohort (Resistant, n = 41; Sensitive, n = 15), and our internal validation cohort (Resistant, n = 17; Sensitive, n = 30). **(B)** Kaplan-Meier curves depicting disease-free survival according to KIF26A expression within an 81-sample cohort. The low and high KIF26A expression groups were differentiated based on the median values detected by the chip. **(C)** Paired cancerous and adjacent non-cancerous samples from the TCGA colorectal cancer dataset (n = 50 pairs). **(D)** The correlation between KIF26A expression and the response of CRC patients to chemotherapy and radiotherapy. The top panel represents statistical analysis using our in-house cohort (n = 44). The bottom panel represents analysis of the TCGA-CRC dataset (n = 69) (NR, no response; PD, progressive disease; SD, stable disease; PR, partial response; CR, complete response). **(E)** The expression levels of KIF26A in the TCGA pan-cancer database were compared with those in the adjacent normal tissues. The TCGA cancer acronyms are listed in the Abbreviations section. **(F)** Kaplan-Meier analyses of progression-free interval (PFI) using the TCGA pan-cancer dataset in patients receiving chemotherapy or radiotherapy or both, based on KIF26A expression levels. A, D and E, *P < 0.05, **P < 0.01, ***P < 0.001 or ****P < 0.0001, determined by Student's *t*-test; *P* value of less than 0.05 indicates a statistical difference. Error bars, mean ± SD.

We further validated the relationship between KIF26A and the sensitivity of patients with CRC to chemoradiotherapy. We investigated several GEO datasets with data on tumor response to chemoradiotherapy 35, 36 and found that KIF26A downregulation was consistently observed in chemoradioresistant CRC, identifying it as a common molecular feature of this phenotype (**Figure 7A**). Analyzing patients with rectal cancer from our prior data revealed a positive relationship between KIF26A expression and DFS [hazard ratio (HR), 0.53; 95% CI = 0.26-0.95, log-rank *P* = 0.044, **Figure 7B**]. ROC curve analysis (area under the curve = 0.815, Supplementary Figure S8A) indicated that KIF26A expression could serve as a prognostic biomarker. Subsequent Kaplan-Meier analyses using this cutoff and a tertile split consistently demonstrated that high KIF26A expression was associated with significantly poorer survival (Supplementary Figure S8B and C), confirming the robustness of its prognostic value. Analysis of TCGA data revealed no significant correlation between KIF26A expression and mutational burden (TMB) or microsatellite instability (MSI) in colorectal cancer, indicating its independent prognostic value (Supplementary Figure S8E). High KIF26A expression in our independent cohort correlated with a higher proportion of treatment-sensitive cases. This finding was consistent with a similar, though non-significant, trend observed in TCGA colorectal cancer data (*P* = 0.229), and was further supported by validation in additional cancer types (ESCA, LIHC, LGG) (**Figure 7D**, and Supplementary Figure S8D). Analysis of TCGA-provided COAD and READ data showed that KIF26A expression in colorectal cancer tissues was markedly downregulated compared to paired normal tissues (**Figure 7C**). Immunohistochemical results provided by the HPA website showed that KIF26A expression was relatively lower in various types of cancers than in normal tissues (Supplementary Figure S8F). Pan-cancer analysis of TCGA data revealed that KIF26A expression is consistently lower in various types of tumors compared to that in normal tissues (**Figure 7E**). Additionally, we examined the expression levels of KIF26A using data from the TCGA Pan-cancer database in a cohort of patients treated with chemotherapy, radiotherapy, or both. Our analysis revealed a positive correlation between KIF26A expression and favorable patient outcomes. As assessed by Kaplan-Meier survival assessment revealed that increased KIF26A was associated with extended progression-free interval (PFI) time in READ, STAD, LUAD, CHOL, PAAD, GBM, KIRC, KICH, LGG (**Figure 7F**) and disease-free interval (DFI) in STAD, CHOL, LIHC and Overall survival (OS) in LUAD, PAAD, LIHC, LAML, KIRC, LGG patients (Supplementary Figures S8G, H). These findings suggest that KIF26A has a substantial impact on colorectal cell proliferation and chemoradiotherapy sensitivity.

## Discussion

Our research highlights a key role of KIF26A in DNA damage repair. Growing evidence implicates members of the kinesin protein family in the DNA damage response and in mediating resistance to therapy. A previous study suggested that KIF5B and KIF13B drive the formation of DNA damage-capturing nuclear envelope tubules (dsbNETs) to promote repair, whereas KIFC3 dismantles them; their balanced activity governs genomic stability and therapeutic response sensitivity in BRCA1-deficient cancers 7. Meanwhile, KIF14 promotes chemotherapy resistance via AKT phosphorylation 8. These studies also highlight kinesins as potential targets for overcoming treatment resistance. However, these mechanisms are largely involved in modulating specialized structures or signaling pathways. In contrast, our study reveals that KIF26A operates through a more direct mechanism. Our discovery that KIF26A directly interacts with Ku70 and modulates NHEJ presents a novel mechanism distinct from the roles of other kinesins implicated in genome maintenance. This direct physical and functional engagement with a core DNA repair complex highlight the unique role of KIF26A.

In this study, we showed that elevated KIF26A expression competitively sequesters Ku70, thereby blocking DNA-PK complex assembly and suppressing NHEJ initiation. Critically, following chemoradiotherapy, we observed increased nuclear localization of KIF26A, which correlates with its enhanced interaction with Ku70. This interaction impairs the repair of chemoradiotherapy-induced DNA damage and sensitizes cancer cells to chemoradiotherapy. The pronounced impact of KIF26A on NHEJ has not been previously reported. This highlights that KIF26A, an atypical kinesin best known for shaping the enteric nervous system, may modulate DNA damage control functions that we are only beginning to appreciate. Collectively, these results suggest that KIF26A may not directly inhibit DNA repair; instead, it may influence the formation of the Ku70/Ku80/DNA-PKcs complex by dynamically translocating to the nucleus and enhancing its sequestration of Ku70 in response to DNA damage. Alterations in the function or expression of proteins in the NHEJ repair pathway can affect sensitivity to chemoradiotherapy. For example, cytoplasmic Ku70 overexpression and nuclear Ku80 loss, correlate with neoadjuvant chemoradiation therapy sensitivity in patients with locally advanced rectal cancer 37. Interactions between the NHEJ repair pathway-critical proteins, such as Ku70/Ku80/DNA-PK, and additional proteins have been reported. For instance, Ku70 binds to VAV1/2, and its interaction with VAV2 affects radiotherapy sensitivity in esophageal cancer 38. Ku70/Ku80 combined with LRRC31 inhibited DNA repair and enhanced the radiosensitivity of brain metastases originating from breast cancer 39. DNA-PKcs interacts with linc00312 to influence nasopharyngeal carcinoma's radiotherapy sensitivity 40. These findings strongly support the main findings of our investigation.

Our data also suggested that chemoradiotherapy modulates KIF26A expression by altering its acetylation levels. After chemoradiotherapy, acetylation of the KIF26A promoter was suppressed, leading to reduced promoter activity and transcriptional repression. To restore the sensitivity of resistant cell lines, we used HDAC inhibitors to block histone deacetylases, thereby elevating KIF26A expression and enhancing chemosensitivity. Various HDACi drugs have been approved for use in clinical cancer therapy. For instance, the FDA has approved multiple HDAC inhibitors including Vorinostat and Romidepsin for treating T-cell lymphomas and multiple myeloma 41-43. However, the efficacy of HDACi in the treatment of solid tumors is limited. Studies have explored combinations of HDACi and chemotherapy. For instance, the combination of Pracinostat and Azacitidine for treating acute myeloid leukemia has been shown to significantly prolong median overall survival 44. Vorinostat and Cisplatin act synergistically to suppress the development of androgen-independent prostate cancer 45. Another study showed that HDACi boosts CD8+ T cell antitumor function, but also induces VEGFa in pro-tumor macrophages, limiting immunotherapy synergy; therefore, a triple regimen of HDACi, anti-angiogenesis, and immunotherapy has been proposed 46. Based on these findings, Vorinostat and other HDACi drugs could serve as effective chemo-radiosensitizers for patients with CRC with low KIF26A expression. This finding provides a rationale for future clinical trials.

What are the translational prospects of our findings? Although no drugs have been specifically designed to upregulate KIF26A, our results indicate that HDAC inhibitors can effectively boost its expression. Multiple HDAC inhibitors, including Vorinostat, Romidepsin, and Panobinostat, have been approved for cancer treatment. Our discovery regarding KIF26A's potential impact on colorectal cancer growth and its response to CRT offers substantial grounds for assessing HDAC inhibitors in conjunction with chemoradiotherapy. Furthermore, pan-cancer analyses revealed that KIF26A was downregulated across multiple tumor types and correlated with key DNA repair molecules, whereas phenotypic validation in lung cancer cell lines confirmed heightened chemo-radiosensitivity upon KIF26A overexpression. Therefore, we propose that combining HDAC inhibitors with standard chemoradiotherapy may be clinically applicable to a broad spectrum of cancers. While our preclinical data support HDAC inhibitor (HDACi) combination therapy for KIF26A-low tumors, its clinical efficacy requires prospective validation. As low KIF26A confers CRT resistance and HDAC inhibition upregulates KIF26A, patients with KIF26A-low tumors represent ideal candidates for this strategy. We therefore propose that future clinical trials of HDACi-CRT stratify patients based on pre-treatment KIF26A expression. This biomarker driven approach enables a precise evaluation of therapeutic efficacy and paves the way for personalized therapy in rectal cancer.

In summary, through analysis and experiments, we found KIF26A to be a crucial factor in chemoradio-resistance. We offer new insights into its role by showing that KIF26A inhibits the organization of the DNA-PK complex and activation of the NHEJ repair pathway. Moreover, HDACi increases KIF26A acetylation and expression, thereby enhancing the chemoradiosensitivity of cells with low KIF26A expression. This finding could be highly significant for the development of effective and precise cancer chemoradiotherapy.

We also observed that tumor cells acquiring chemoradiotherapy resistance were also accompanied by enhanced migration and invasion abilities. This phenomenon suggests that histone deacetylase inhibitors such as Vorinostat can effectively reverse this migratory and invasive phenotype. We speculate that treatment resistance in tumor cells is not an isolated characteristic, but rather a part of their overall malignant progression. Targeting key epigenetic regulatory nodes in this process (such as HDAC) not only holds promise for overcoming treatment resistance, but may also effectively inhibit tumor metastasis and dissemination, with significant clinical translational implications. While our current investigation focused on the role of H3K9 acetylation in upregulating KIF26A, the involvement of other specific histone acetylation sites requires further study. Our future studies will further explore this area in depth to uncover more precise molecular mechanisms.

Although research on chemoradiotherapy resistance in rectal cancer has progressed, numerous issues remain to be addressed. Future studies should deepen our understanding of resistance mechanisms and seek effective therapeutic targets and strategies. Concurrently, enhancing multidisciplinary collaboration is essential to expedite clinical translation, thereby enhancing the therapeutic efficacy and overall well-being of individuals with colorectal cancer or other solid tumors.

## Conclusion

Our study has elucidated the mechanisms by which KIF26A affects chemoradiotherapy sensitivity and has demonstrated that the combination of HDAC inhibitors (HDACi) with chemoradiotherapy may represent a promising clinical strategy for the treatment of colorectal cancer.

## Supplementary Material

Supplementary figures.

Supplementary tables 1-4.

Supplementary table 5.

Supplementary table 6.

## Data Availability

Correlation, enrichment, regression, and gene expression profiling were performed utilizing data from TCGA CRC and Pan-cancer datasets (https://portal.gdc.cancer.gov), CellMiner CDB (https://discover.nci.nih.gov/cellminercdb/), and GDSC (https://www.cancerrxgene.org/). Gene Ontology (GO) and Gene Set Enrichment Analysis (GSEA) were conducted with the R package cluster Profiler. Raw RNA-seq data for KIF26A-OE RKO cells and vector controls have been deposited in the National Genomic Data Center (https://ngdc.cncb.ac.cn/gsub/) under accession PRJCA044798. These data are available upon reasonable request to the corresponding author. Kaplan-Meier analysis was performed using data from Huang *et al* 10 and TCGA Pan-cancer datasets. Data from external sources or prior studies are cited in the text and figure legends.

## Abbreviations

KIF26A: Kinesin Family Member 26A
CRC: Colorectal Cancer
CRT: Chemoradiotherapy
DNA-PK: DNA-Dependent Protein Kinase
NHEJ: Non-Homologous End Joining
HDACi: Histone Deacetylase Inhibitor
DSBs: Double-Strand Breaks
DNA-PKcs: DNA-Dependent Protein Kinase Catalytic Subunit
DNA-PK catalytic subunit: DNA-Dependent Protein Kinase Catalytic Subunit
dsbNETs: DSB-capturing nuclear envelope tubules
TRG: Tumor Regression Grade
DFS: Disease-Free Survival
HR: Homologous Recombination
GEO: Gene Expression Omnibus
TCGA: The Cancer Genome Atlas
GO: Gene Ontology
GSEA: Gene Set Enrichment Analysis
IP-WB: Immunoprecipitation Western Blot
CoIP-WB: Co-Immunoprecipitation Western Blot
IR: Ionizing Radiation
HATs: Histone Acetyltransferases
HDACs: Histone Deacetylases
TSA: Trichostatin A
DMSO: Dimethyl Sulfoxide
CR: Complete Response
PR: Partial Response
SD: Stable Disease
PD: Progression Disease
NR: No Response
ACC: Adrenocortical carcinoma
BLCA: Bladder urothelial carcinoma
BRCA: Breast invasive carcinoma
CESC: Cervical squamous cell carcinoma and endocervical adenocarcinoma
CHOL: Cholangiocarcinoma
COAD: Colon adenocarcinoma
CRC: Colon adenocarcinoma/Rectum adenocarcinoma
DLBC: Diffuse large B-cell lymphoma
ESCA: Esophageal carcinoma
GBM: Glioblastoma multiforme
HNSC: Head and neck squamous cell carcinoma
KICH: Kidney chromophobe
KIRP: Kidney renal papillary cell carcinoma
KIRC: Kidney renal clear cell carcinoma
LAML: Acute Myeloid Leukemia
LGG: Lower-grade glioma
LIHC: Liver hepatocellular carcinoma
LUAD: Lung adenocarcinoma
LUSC: Lung squamous cell carcinoma
MESO: Mesothelioma
OV: Ovarian serous cystadenocarcinoma
PAAD: Pancreatic adenocarcinoma
PCPG: Pheochromocytoma and paraganglioma
PRAD: Prostate adenocarcinoma
READ: Rectum adenocarcinoma
SARC: Sarcoma
SKCM: Skin cutaneous melanoma
STAD: Stomach adenocarcinoma
TGCT: Testicular germ cell tumor
THCA: Thyroid carcinoma
THYM: Thymoma
UCEC: Uterine corpus endometrial carcinoma
UCS: Uterine carcinosarcoma
UVM: Uveal melanoma
