# Supplementary material for: Kinesin Family Member 26A Disrupts DNA-Dependent Protein Kinase Complex Formation to Enhance Chemoradiotherapy Sensitivity in Colorectal Cancer
Source: Int J Biol Sci. 2026 Mar 17;22(7):3411–31. doi: 10.7150/ijbs.127218 (PMC13085882; doi:10.7150/ijbs.127218)
Supplement: Supplementary file 1 — Supplementary figures. [file ijbsv22p3411s1.pdf]

Supplementary Material
Supplementary Figures and Legends:

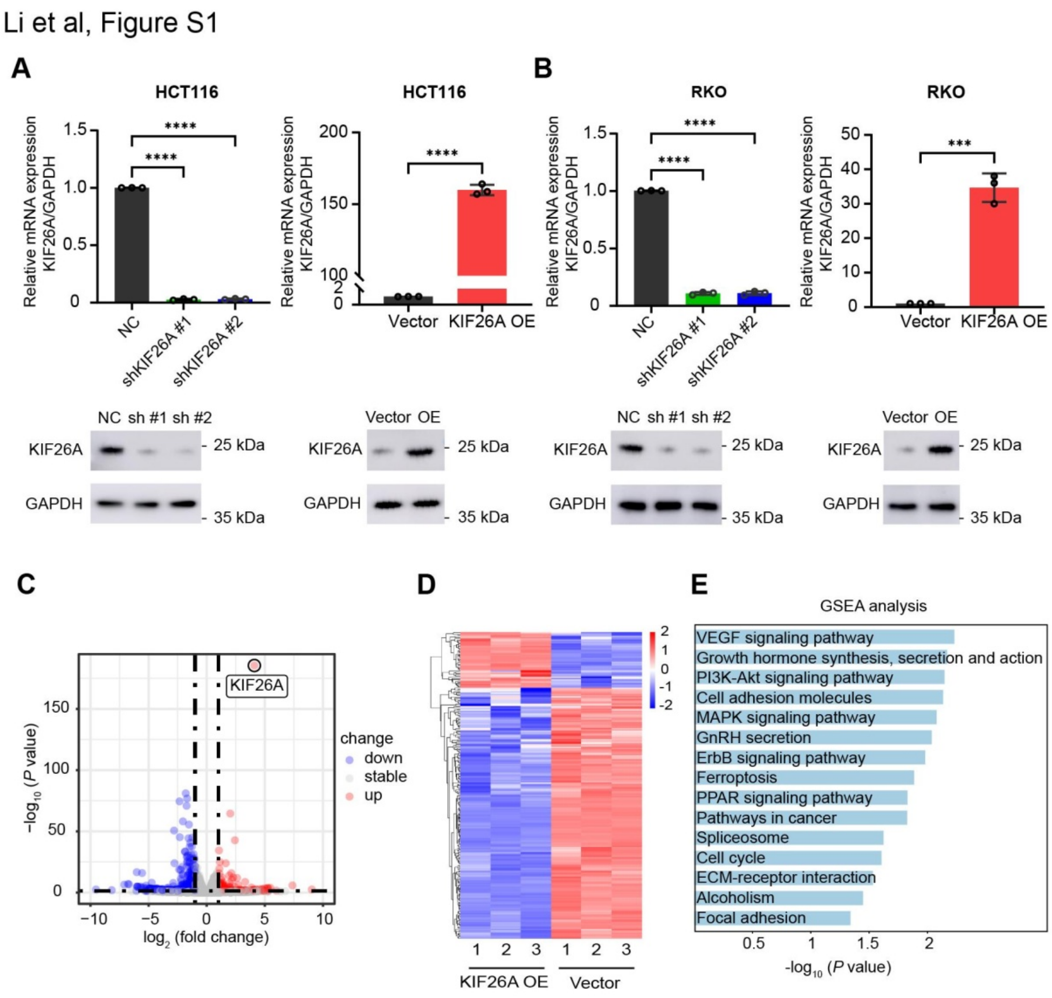

Figure S1. KIF26A functions as a DNA damage response protein. Related to Figure 1.

A. mRNA expression of KIF26A in KIF26A-sh and KIF26A-OE HCT116 quantified by RT-qPCR and Western blot (n = 3).

B. mRNA expression of KIF26A in KIF26A-sh and KIF26A-OE RKO quantified by RT-qPCR and Western blot (n = 3).

C-D. (C) Volcano plots and heatmap (D) showing genes upregulated (red, fold change > 2; $P < 0.05$) or downregulated (blue, fold change < 0.5; $P < 0.05$) in RKO cells overexpressing KIF26A.

Gene set enrichment analysis (GSEA) of differentially expressed genes showed several pathways, including the PI3K-AKT signaling pathway and cell adhesion molecules.

A and B, ***$P < 0.001$ or ****$P < 0.0001$, determined by Student's *t*-test and one-way ANOVA; *P* value of less than 0.05 indicates a statistical difference. Error bars, mean ± SD. A-B, three biological independent replicates were performed.

Li et al, Figure S2

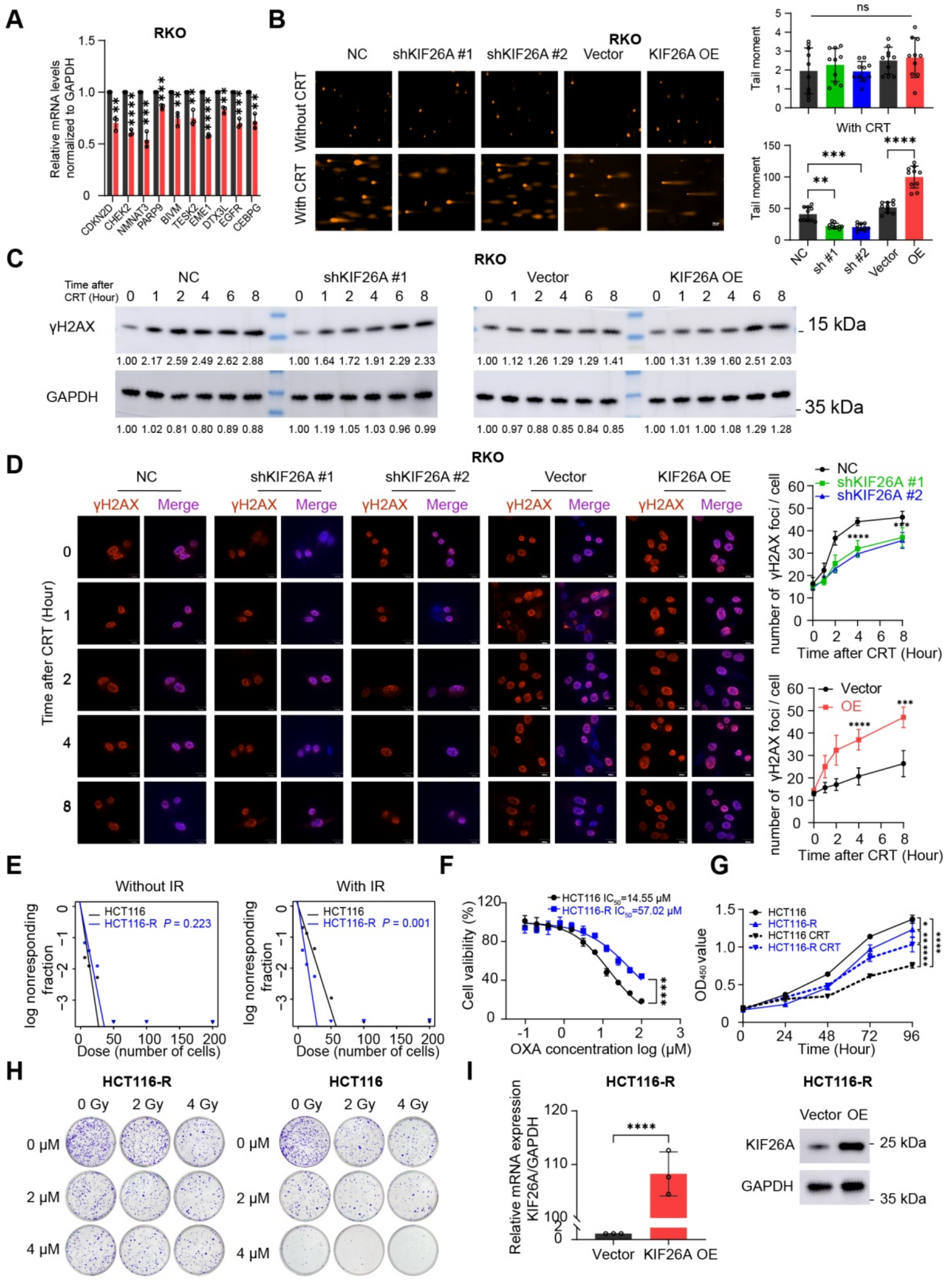

Figure S2. KIF26A inhibits DNA damage repair. Related to Figure 2.

A. Verification of the expression changes of the differentially expressed genes in the DNA repair pathway in RKO cell (n = 3).

B. DNA damage in KIF26A-modulated RKO cells after CRT was assayed by comet assay (n = 10). Scale bars, 100 μm.

C, D. DNA double-strand breaks expressed by γ-H2AX level in KIF26A knockdown or overexpressing RKO cells treated with or without CRT. (C) Shows γ-H2AX by western blot analysis. (D) Shows images of γ-H2AX foci in cells at various time

points of IR as indicated (n = 3). Scale bars, 10 μm.

E. Cell survival fractions via extreme limiting dilution analysis in HCT116 parental cells (HCT116) and HCT116 chemoradiotherapy-resistant cells (HCT116-R) with or without IR (4 Gy).

F. IC_{50} values of Oxaliplatin(OXA)in HCT116 parental cells (HCT116) and HCT116 chemoradiotherapy-resistant cells (HCT116-R) (n = 6).

G. CCK8 assay showed the proliferation ability of HCT116 parental cells (HCT116) and HCT116 chemoradiotherapy-resistant cells (HCT116-R) (n = 6). CRT group treated with radiation (4 Gy) and chemotherapy (1 μg/mL of Oxaliplatin); Control group treated with the same volume of DMSO as the CRT group and without radiation.

H. The colony formation assay showed the proliferation ability of HCT116 parental cells (HCT116) and HCT116 chemoradiotherapy-resistant cells (HCT116-R) under different doses of radiotherapy and chemotherapy drugs (n = 3).

I. mRNA expression of KIF26A in KIF26A-OE HCT116-R quantified by RT-qPCR and Western blot (n = 3).

A, B, D, F, G, I, *$P < 0.05$, **$P < 0.01$, ***$P < 0.001$ or ****$P < 0.0001$, A, E, F, I, determined by Student's *t*-test; B, D, G, determined by one-way ANOVA. *P* value of less than 0.05 indicates a statistical difference. Error bars, mean ± SD. For all panels, three biological independent replicates were performed.

Li et al, Figure S3

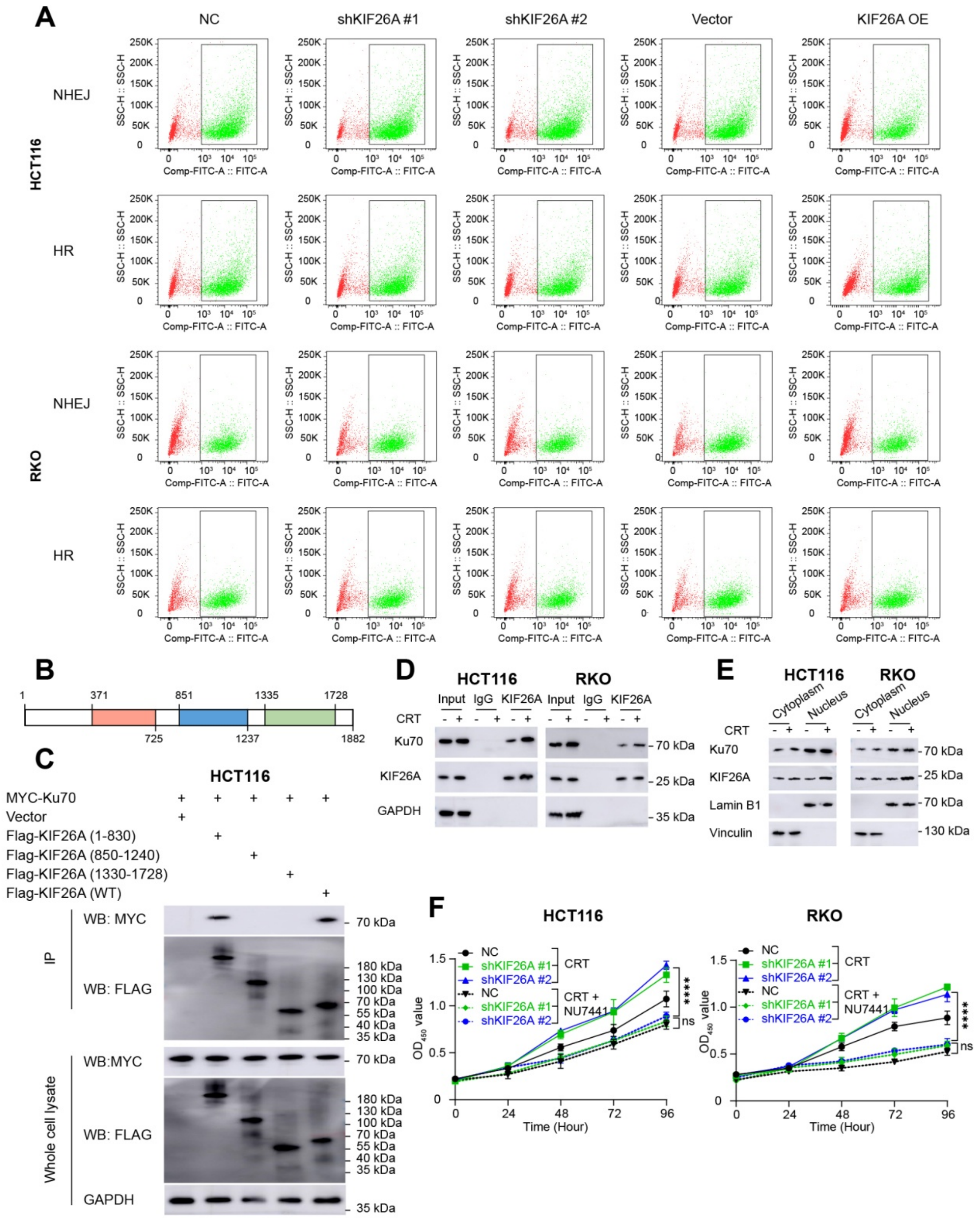

Figure S3. KIF26A inhibits the NHEJ repair pathway by binding to Ku70. Related to Figure 3.

A. Flow cytometry analysis of KIF26A knockdown or overexpression effects on NHEJ and HR pathways. HCT116 and RKO cells were transfected with pEJ5-GFP or DR-GFP. After 24 h, cells were transfected with I-SceI. GFP expression was quantified by flow cytometry 24 hours later, with example gating.

B. Analysis of the architecture of KIF26A.

C. Immunoblot analysis of Myc-tagged Ku70 co-immunoprecipitated with FLAG-tagged full-length (WT) KIF26A and its truncated variants.
D. In HCT116 and RKO cells, Western blot probed KIF26A and Ku70 proteins immunoprecipitated via anti-KIF26Aantibody, with IgG as the control.
E. Analysis of KIF26A and Ku70 levels in cytoplasmic and nuclear fractions of HCT116 cells by Western blot.
F. CCK8 assay showed the proliferation ability of HCT116 and RKO control (NC) and KIF26A-knockdown (sh) cells following CRT treatment with or without NU7441 (n = 6).

A, C-E three independent replicates were performed. CRT group treated with radiation (8 Gy) and chemotherapy (2 μg/mL of Oxaliplatin). F. Combination groups were treated with NU7441 (1 μM) for 2 hours prior to CRT treatment. A and C-F, three biological independent replicates were performed.

Li et al, Figure S4

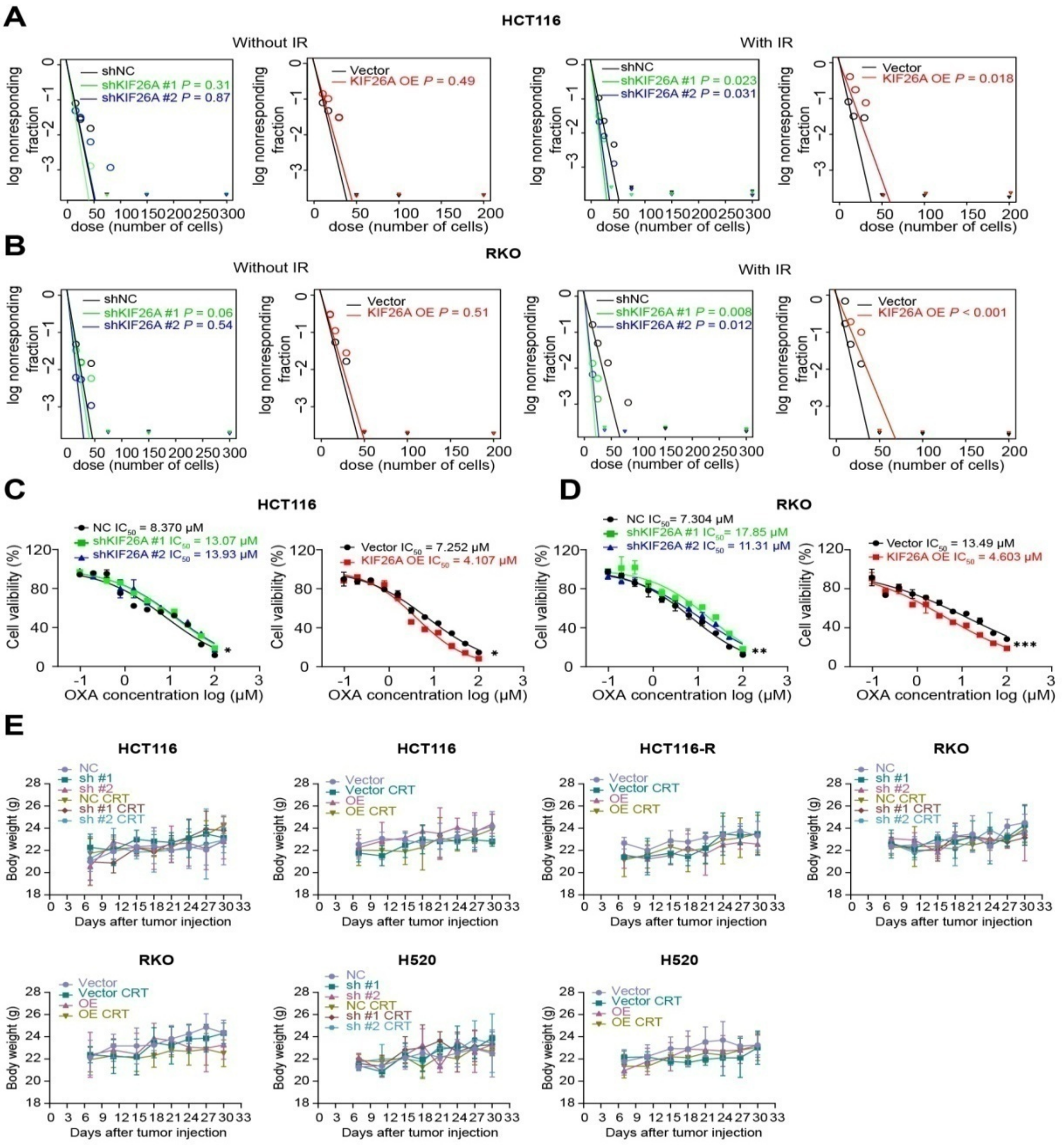

Figure S4. KIF26A increases chemoradiotherapy sensitivity. Related to Figure 4.

A-B. Cell survival fractions via extreme limiting dilution analysis in KIF26A-knockdown and KIF26A-OE HCT116 (A) and RKO (B) cells with or without IR (4 Gy).

C-D. IC_{50} values of Oxaliplatin (OXA) in KIF26A-knockdown and KIF26A-OE HCT116 (C) and RKO (D) cells (n = 6).

E. In vivo toxicity monitoring. Bodyweight growth curves of control and KIF26A-knockdown or KIF26A-overexpression tumors in HCT116, HCT116-R, RKO and H520 cells with or without CRT treatment (n = 4-5 per group).

A-D, *$P < 0.05$, **$P < 0.01$, ***$P < 0.001$, A-D, determined by Student's t-test and one-way ANOVA. P value of less than 0.05 indicates a statistical difference. Error bars, mean ± SD. For all panels, three biological independent replicates were performed.

Li et al, Figure S5

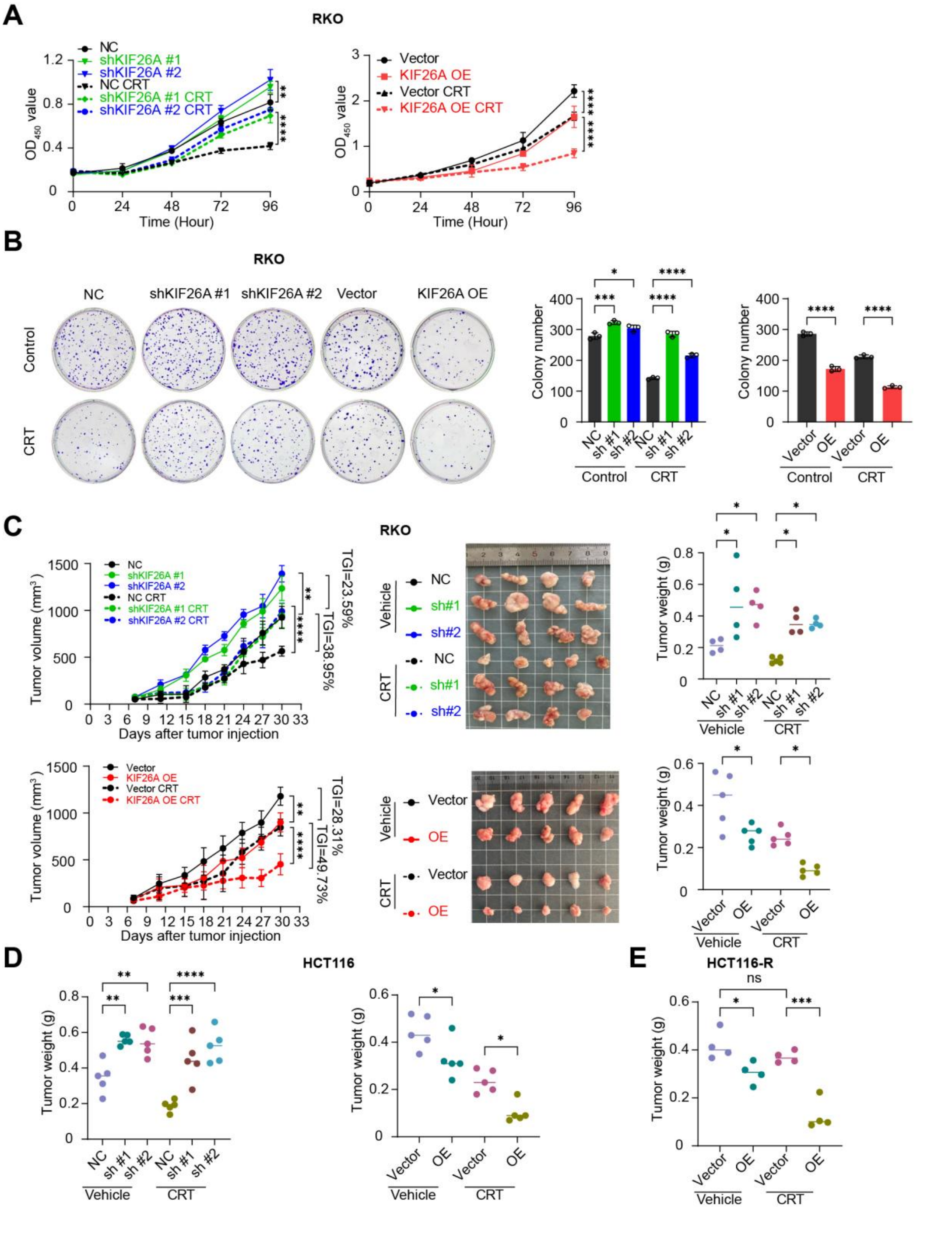

Figure S5. KIF26A increases chemoradiotherapy sensitivity. Related to Figure 4.

A. CCK8 assay showed the proliferation ability of RKO cells following KIF26A knockdown or overexpression (n = 6).

B. The colony formation assay showed the proliferation ability of RKO cells following KIF26A knockdown or overexpression (n = 3).

C. RKO cells were subcutaneously injected into NSG mice and then treated with radiation (4 Gy) and Oxaliplatin (5 mg/kg) or not at the indicated time points (n = 4-5 per group). Tumor growth curves (left), excised tumor images (middle) and

tumor weight (right) of control and KIF26A-knockdown tumors, KIF26A-overexpression tumors in RKO cells with or without CRT treatment (n = 5 per group). TGI, percentage of tumor growth inhibition.

D. Tumor weight of control and KIF26A-knockdown tumors, KIF26A-overexpression tumors in HCT116 cells.

E. Tumor weight of control and KIF26A-overexpression tumors in HCT116-R cells.

A and B, CRT group treated with radiation (4 Gy) and chemotherapy (1 μg/mL of Oxaliplatin); Control group treated with the same volume of DMSO as the CRT group and without radiation. A-C, $^{**}P < 0.01$, $^{***}P < 0.001$ and $^{****}P < 0.0001$; determined Student's *t*-test and one-way ANOVA; *P* value of less than 0.05 indicates a statistical difference. Error bars, mean ± SD. A and B, three biological independent replicates were performed.

Li et al, Figure S6

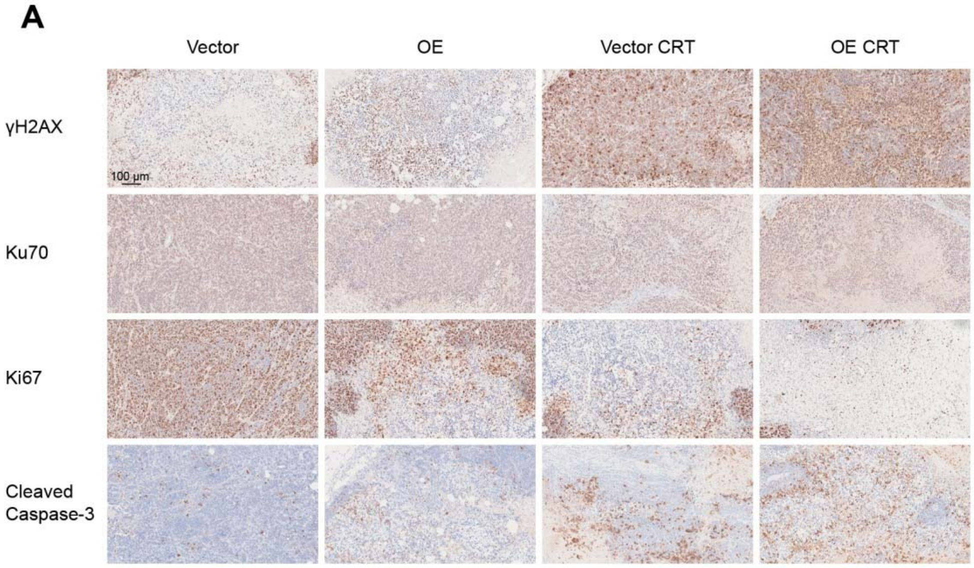

Figure S6. Immunohistochemistry of HCT116 xenografts. Related to Figure 4.

A. Staining for γ-H2AX, Cleaved Caspase-3, KU70, and Ki67 in tumors. Scale bar: 100 μm. Three independent biological replicates were performed.

Li et al, Figure S7

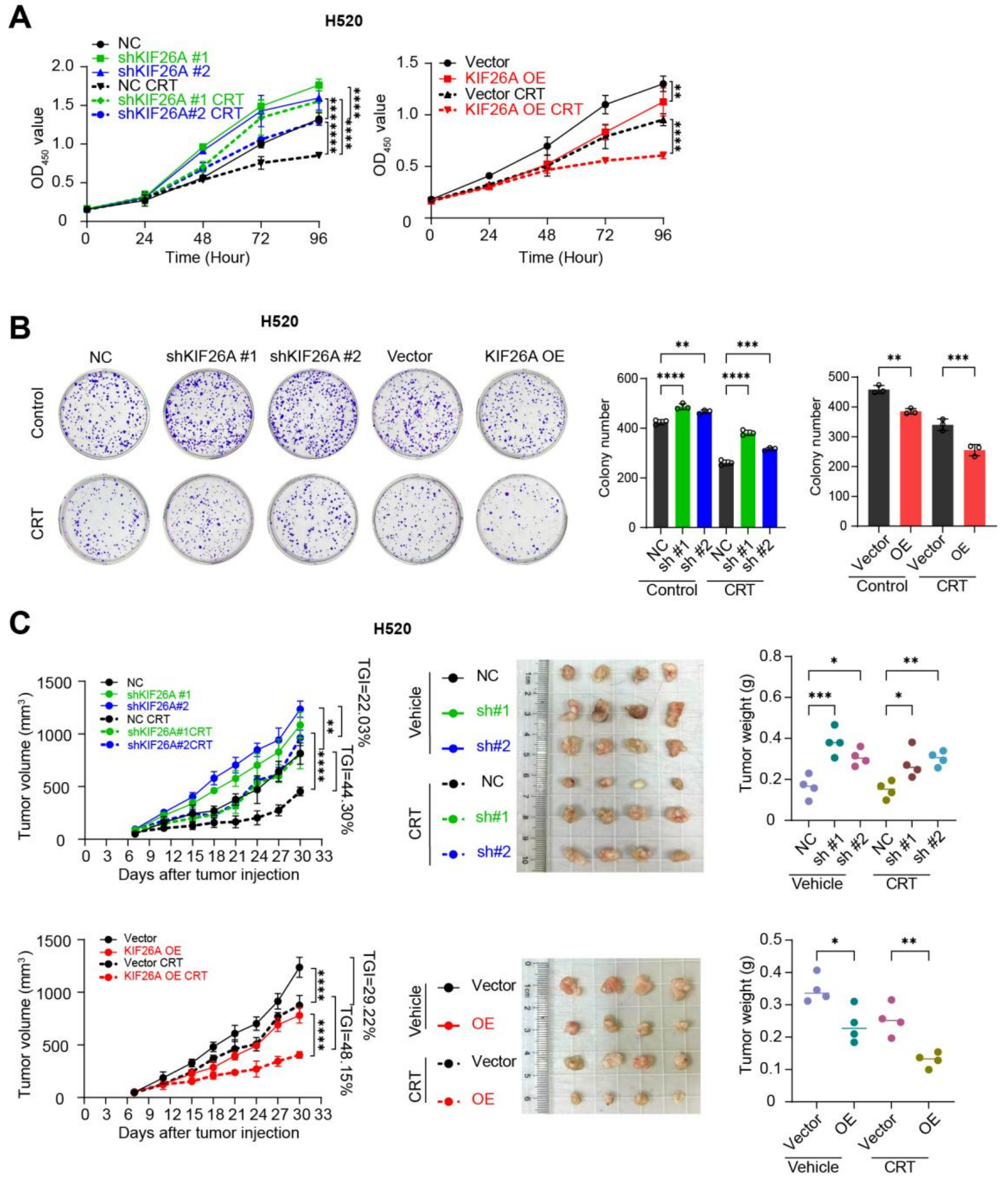

Figure S7. KIF26A increases chemoradiotherapy sensitivity. Related to Figure 4.

A. CCK8 assay showed the proliferation ability of H520 cells following KIF26A knockdown or overexpression (n = 6).

B. The colony formation assay showed the proliferation ability of H520 cells following KIF26A knockdown or overexpression (n = 3).

C. H520 cells were subcutaneously injected into NSG mice and then treated with radiation (4 Gy) and Oxaliplatin (5 mg/kg) or not at the indicated time points (n = 4 per group). Tumor growth curves (left), excised tumor images (middle) and

tumor weight (right) of control and KIF26A-knockdown tumors, KIF26A-overexpression tumors in H520 cells with or without CRT treatment (n = 4 per group). TGI, percentage of tumor growth inhibition.

A and B, CRT group treated with radiation (4 Gy) and chemotherapy (1 μg/mL of Oxaliplatin); Control group treated with the same volume of DMSO as the CRT group and without radiation. A-C, $^{**}P < 0.01$, $^{***}P < 0.001$ and $^{****}P < 0.0001$; determined Student's *t*-test and one-way ANOVA; *P* value of less than 0.05 indicates a statistical difference. Error bars, mean ± SD. A and B, three biological independent replicates were performed.

Li et al, Figure S8

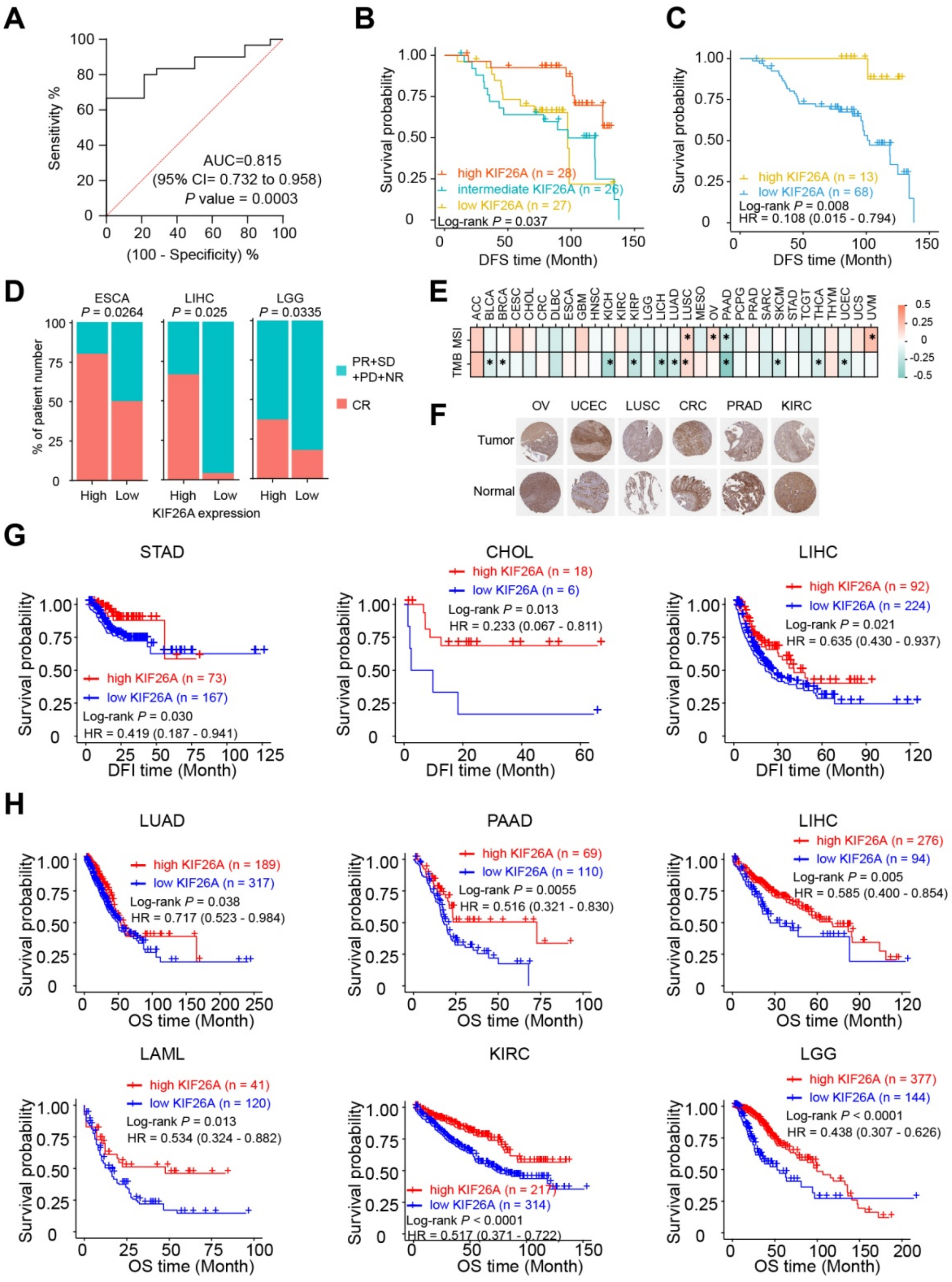

Figure S8. Reduced KIF26A expression informs poor prognosis of patients to chemoradiotherapy. Related to Figure 7.

A. Receiver operating characteristic (ROC) curve for evaluating the prognostic performance of KIF26A expression. The optimal cutoff value (7.44) was determined by maximizing the Youden index.

B. Kaplan-Meier survival curves based on tertile stratification of KIF26A expression. Patients were divided into low, intermediate and high expression groups of equal size.

C. Kaplan-Meier survival curves based on the optimal ROC-determined cutoff for KIF26A expression. Patients were stratified into low- and high-expression groups using this cutoff.

D. The correlation between KIF26A expression and the response of CRC patients to chemotherapy and radiotherapy. The left panel represents analysis of the TCGA-ESCA dataset (n = 58). The middle panel represents analysis of the TCGA-LIHC dataset (n = 27). The right panel represents analysis of the TCGA-LGG dataset (n = 217)(NR, no response; PD, progressive disease; SD, stable disease; PR, partial response; CR, complete response).

E. Correlation analysis between KIF26A expression and genomic instability markers using the TCGA pan-cancer dataset in patients receiving chemotherapy or radiotherapy or both.

F. Immunohistochemistry of cancer and normal tissues from the Human Protein Atlas.

G. Kaplan-Meier analyses of disease-free interval (DFI) using the TCGA pan-cancer dataset in patients receiving chemotherapy or radiotherapy or both, based on KIF26A expression levels. The TCGA cancer acronyms are listed in the Abbreviations section.

H. Kaplan-Meier analyses of overall survival (OS) using the TCGA pan-cancer dataset in patients receiving chemotherapy or radiotherapy or both, based on KIF26A expression levels.
