# Supplementary material for: Kinesin Family Member 26A Disrupts DNA-Dependent Protein Kinase Complex Formation to Enhance Chemoradiotherapy Sensitivity in Colorectal Cancer
Source: Int J Biol Sci. 2026 Mar 17;22(7):3411–31. doi: 10.7150/ijbs.127218 (PMC13085882; doi:10.7150/ijbs.127218)
Supplement: Supplementary file 2 — Supplementary tables 1-4. [file ijbsv22p3411s2.pdf]

Supplementary Tables

Table S1. Patient characteristics by KIF26A expression group

Variable	High KIF26A<br>N = 40	Low KIF26A<br>N = 41	*P* value*
Age			0.3
< 54	22(55%)	18(44%)	
≥ 54	18(45%)	23(56%)	
Gender			0.4
Male	25 (63%)	29 (71%)	
Female	15 (38%)	12 (29%)	
Tumor Stage			0.2
Stage II	6 (15%)	13 (32%)	
Stage III	34 (85%)	28 (68%)	
Karnofsky Performance Status			>0.9
80-90	17 (43%)	17 (41%)	
90-100	23 (58%)	24 (59%)	

*Pearson's Chi-squared test

Table S2. Short hairpin RNA sequences

Gene	Short hairpin RNA sequences (5' → 3')
NC	TTCTCCGAACGTGTCACGT
shKIF26A#1	GCATCGGGAAGGTGAAGGTTA
shKIF26A#2	GCATCAATGATGAGTTTGACG

Table S3. Small interfering RNA sequences

Gene	Small interfering RNA sequence (5' →3')
NC	UUCUCCGAACGUGUCACGUTT
JunB	#1: CACGACTACAAACTCCTGAAA
HDAC1	#1: CGGUCAUGUCCAAAGUAAUTT
HDAC2	#1: UGUGAAGUUAAACCGACAATT
HDAC3	#1: AAUCAGAACUCACGCCAGUTT
HDAC4	#1: CGAGCACUGUGGUUUACAATT
HDAC5	#1: CGGGUUUGAUGCUGUUGAATT
HDAC6	#1: GCAAUGGAAGAAGACCUAATT

Table S4. Primers used for quantitative real time PCR analysis

Quantitative real time PCR	Sequence (5' →3')
AKT1-F	TCCTCCTCAAGAATGATGGCA
AKT1-R	GTGCGTTCGATGACAGTGGT
BEND3-F	CAGGCGAGTTTGCCGTCTT
BIVM-F	TCTGGAAATGGTGAGCACAAAT
BIVM-R	CCGTGTATGAGTCACTGGACA

BND3-R	CTCCGTGTAGTTGCGGATGAT
BRCA2-F	TGCCTGAAAACCAGATGACTATC
BRCA2-R	AGGCCAGCAAACTTCCGTTTA
BRPF3-F	AGCTTCCGTATGGTGGACTCA
BRPF3-R	AGGTGGCTTCTCAATGTAGCG
CDKN2D-F	AGTCCAGTCCATGACGCAG
CDKN2D-R	ATCAGGCACGTTGACATCAGC
CEBPG-F	ACTCCAGGGGTGAACGGAAT
CEBPG-R	CATGGGCGAACTCTTTTTGCT
CHEK2-F	TGAGAACCTTATGTGGAACCCC
CHEK2-R	ACAGCACGGTTATACCCAGC
CTCF-F	CAGTGGAGAATTGGTTCGGCA
CTCF-R	CTGGCGTAATCGCACATGGA
DTX3L-F	AGCACCAAATACTTGTTGACGA
DTX3L-R	CACCAGACGGTGTTTCTGCTT
EGFR-F	TTGCCGCAAAGTGTGTAACG
EGFR-R	GTCACCCCTAAATGCCACCG
EME1-F	TCTGAGGAGTTGCCAACATTTG
EME1-R	GGCTTCACAATCTGAGATGTCAA
FGFR1-F	GGCTACAAGGTCCGTTATGCC
FGFR1-R	GATGCTGCCGTACTCATTCTC
FLT3LG-F	TGGAGCCCAACAACCTATCTC
FLT3LG-R	ACGGATTTTGACAGCGAAGTC
GAPDH-F	AACGGATTTGGTCGTATTGGG
GAPDH-R	TGATTTTGGAGGGATCTCGC
GDPD5-F	GATCGTGGCAGGACAGTTCG
GDPD5-R	GGGCCGAGGTCTTTCTTCT
HTRA2-F	CGCGAGGTCCCTATCTCGAA
HTRA2-R	GCTTAGCAGTCTCACACGGA
ING5-F	CGGTGAAGACGCTGTCTCC
ING5-R	TGCACTTTGTCGTCACTGTATTC
JUNB-F	TCCAAGTGCCGAAAAAGGAAG
JUNB-R	CGAGTTCTGAGCTTTCAAGGT
KAT5-F	AACAAACGTCTGGATGAATGGG
KAT5-R	AGGAAGTCCGTTCTTAGTGGG
KIF26A-F	CGGTGACCCCGATTACTCCT
KIF26A-R	CAGGCGGACCTTCGTTGTC
NMNAT3-F	GAGTAGGTCACGACCCAAAAG
NMNAT3-R	TCGCCTGATGTATGTGGCAC
NR4A3-F	GGTCGTCTGCCTTCCAAAC
NR4A3-R	GCTCGGACAAGGGCATTCA
PARP9-F	TCTGATGGGATTCAACGTGGA
PARP9-R	TTCCTGGGCTGATAATTTCTGTG
PDK1-F	GAGAGCCACTATGGAACACCA

PDK1-R	GGAGGTCTCAACACGAGGT
PER1-F	GCCAACCAGGAATACTACCAGC
PER1-R	GTGTGTACTCAGACGTGATGTG
POLE3-F	GGCCCGAGGACCTAAACCT
POLE3-R	ATGTGGCGTACAGCACGAAG
POLE4-F	GAGGGACCTGCTGGGGAG
POLE4-R	AGCGCAACAGTAGGCATCTT
PTEN-F	TTTGAAGACCATAACCCACCAC
PTEN-R	ATTACACCAGTTCGTCCCTTTC
PTGS2-F	CTGGCGCTCAGCCATACAG
PTGS2-R	CGCACTTATACTGGTCAAATCCC
RHOB-F	CTGCTGATCGTGTTCAGTAAGG
RHOB-R	TCAATGTCGGCCACATAGTTC
STOX1-F	TTGGGTGAAGTTCTTTGCTGT
STOX1-R	TTTCATCAAACGCTCCAAAAGTG
TAF6L-F	GGCCACGCAGAATAGCTCTC
TAF6L-R	GTGATCCGTAACCACACACAG
TESK2-F	GAAGTGTTCAAGGTACGACACC
TESK2-R	ATGTTTGCCCGGTTACTGCTC
TRIM21-F	TCAGCAGCACGCTTGACAAT
TRIM21-R	GGCCACACTCGATGCTCAC
TRIM32-F	TTGCAGCAAGATTACCCGCAT
TRIM32-R	CAGGACCGACACATGAGCA
